# Patient Perspectives About Decisions to Share Medical Data and Biospecimens for Research

**DOI:** 10.1001/jamanetworkopen.2019.9550

**Published:** 2019-08-21

**Authors:** Jihoon Kim, Hyeoneui Kim, Elizabeth Bell, Tyler Bath, Paulina Paul, Anh Pham, Xiaoqian Jiang, Kai Zheng, Lucila Ohno-Machado

**Affiliations:** 1 Department of Biomedical Informatics, UC San Diego Health, University of California, San Diego, La Jolla; 2 School of Nursing, Duke University, Durham, North Carolina; 3 Office of Graduate Studies and Research, California State University, San Marcos; 4 School of Biomedical Informatics, University of Texas Health Science Center, Houston; 5 Department of Informatics, University of California, Irvine; 6 Division of Health Services Research and Development, Veterans Affairs San Diego Healthcare System, La Jolla, California

## Abstract

**Importance:**

Patients increasingly demand transparency in and control of how their medical records and biospecimens are shared for research. How much they are willing to share and what factors influence their sharing preferences remain understudied in real settings.

**Objectives:**

To examine whether and how various presentations of consent forms are associated with differences in electronic health record and biospecimen sharing rates and whether these rates vary according to user interface design, data recipients, data and biospecimen items, and patient characteristics.

**Design, Setting, and Participants:**

For this survey study, a data and biospecimen sharing preference survey was conducted at 2 academic hospitals from May 1, 2017, to September 31, 2018, after simple randomization of patients to 1 of 4 options with different layout and formats of indicating sharing preferences: opt-in simple, opt-in detailed, opt-out simple, and opt-out detailed.

**Interventions:**

All participants were presented with a list of data and biospecimen items that could be shared for research within the same health care organization or with other nonprofit or for-profit institutions. Participating patients were randomly asked to select the items that they would share (opt-in) or were asked to select items they would not share (opt-out). Patients in these 2 groups were further randomized to select only among 18 categories vs 59 detailed items (simple vs detailed form layout).

**Main Outcomes and Measures:**

The primary end points were the percentages of patients willing to share data and biospecimen categories or items.

**Results:**

Among 1800 eligible participants, 1246 (69.2%) who completed their data sharing survey were included in the analysis, and 850 of these patients (mean [SD] age, 51.1 [16.7] years; 507 [59.6%] female; 677 [79.6%] white) responded to the satisfaction survey. A total of 46 participants (3.7%) declined sharing with the home institution, 352 (28.3%) with nonprofit institutions, and 590 (47.4%) with for-profit institutions. A total of 836 (67.1%) indicated that they would share all items with researchers from the home institution. When comparing opt-out with opt-in interfaces, all 59 sharing choice variables (100%) were associated with the sharing decision. When comparing simple with detailed forms, only 14 variables (23.7%) were associated with the sharing decision.

**Conclusions and Relevance:**

The findings suggest that most patients are willing to share their data and biospecimens for research. Allowing patients to decide with whom they want to share certain types of data may affect research that involves secondary use of electronic health records and/or biosamples for research.

## Key Points

**Question:**

Do patient decisions about sharing their electronic health records and biospecimens for research vary according to health care institution, data or biospecimen item, patient characteristics, data recipient, and format in which consent choices are presented?

**Findings:**

In this survey study of 1246 patients who completed a data and biospecimen sharing survey after being randomly assigned to 1 of 4 options with different layout and formats of indicating sharing preferences, patient preference for sharing compared with no sharing was significantly higher after controlling for covariates when presented with the opt-out compared with the opt-in format. The form layout (detailed vs simple) was not associated with the sharing decision.

**Meaning:**

The findings suggest that many patients may be willing to share data and biospecimens for research and that researchers’ affiliations, the design of consent forms, and patient age and health literacy are associated with patient sharing decisions.

## Teaser

This survey study examines the association between various presentations of consent forms and differences in electronic health record and biospecimen sharing rates among patients.

## Introduction

Use of personal data without explicit user consent has recently put technology companies in the public spotlight.^1,2,3,4^ In contrast, it appears that fewer concerns have been raised regarding the use of medical records and biospecimens,^5^ which are also sensitive, for secondary use purposes such as research. It is unclear whether this relative lack of concern is because patients are generally unaware that their deidentified records are being made available to researchers,^6^ their lack of knowledge that anonymized records can be traced back to individuals,^7,8^ or simply because there have not been many widely publicized incidents to date.^9^

Current laws and regulations require health care institutions to comply with a minimally necessary standard in sharing patient medical records and biospecimens for research.^10^ Some legislation^11,12^ regulates research reuse of patient medical records and biospecimens so that health care institutions can allow deidentified data sharing and identified data sharing (as long as proper institutional review board approvals have been obtained) unless the patient explicitly declines the use of data and biospecimens for purposes other than direct patient care. This all-or-nothing option is problematic because, alerted by recent high-profile cases, the increasing awareness among the general public regarding inappropriate reuse of personal data without explicit user consent^13^ may markedly change patients’ attitudes toward secondary use activities that involve their medical records and biospecimens (ie, patients may start denying research access to all of their data and biospecimens, even if they are willing to share most of their data and biospecimens or to share them only with certain types of institutions).^14,15^ The regulatory landscape is also changing; for example, in the United States as of September 23, 2013,^16^ newly enrolled patients, who need to sign a Health Insurance Portability and Accountability Act (HIPAA) authorization, must opt in to allow the use of their personal health information for optional substudies and future secondary use. In the European Union, the General Data Protection Regulation^17^ implemented in 2018 requires that patients provide consent for clinical data use for research. These issues point to a critical need to better understand patients’ sharing preferences.

Surveys using hypothetical scenarios have been conducted,^18,19,20^ but there has been a paucity of research studies involving electronic health record (EHR) and biospecimen sharing preferences applied in real settings.^21^ Tiered consent (ie, breaking the record into smaller units in a consent form and allowing partial use of the EHR) is not routinely available in practice today, limiting patients’ rights and participation in how their health data are being shared, while there is increasing evidence that what patients want to be asked^6^ and what they consider to be sensitive varies. In California, patient’s specific permission to share mental health, substance abuse, HIV status, and genetic information is required in HIPAA authorization forms, but no other items are specified.^22^ In many states, there is no requirement for patient’s specific permission for sharing these types of data items.^10^

Our study assessed patients’ preferences toward sharing specific data items in their EHRs and biospecimens with different types of researchers. We hypothesized that there would be different decisions for sharing, depending on researchers’ affiliations, patient characteristics, and the user interface design format of the consent form in which data sharing preferences were elicited. In this study, we randomly assigned patients to 1 of 4 types of preference elicitation forms to examine whether the form layout and opting-in or opting-out method were associated with patients’ sharing preferences.

## Methods

### Study Design and Population

For this survey study, patients were recruited from 2 academic medical centers in Southern California. They were approached by email invitation or in person in the waiting area of 10 adult outpatient clinics. Inclusion criteria were age of 18 years or older, being a patient at either academic medical center, and ability to read English or Spanish. Among 1800 eligible participants, 1582 signed a consent form to participate in this study. Of these, 1246 (69.2%) completed their data sharing survey and were included in the analysis, and 850 (68.2%) of these responded to the satisfaction survey. Although it was preferred that all research activities be conducted through the research website, the study provided an option to allow patients who did not have easy access to the internet to participate using paper forms. Preference elicitation and surveys were conducted from May 1, 2017, to September 31, 2018. Written informed consent was obtained from the web portal immediately after sign-up for online users and via a paper form for other users. Data were deidentified for the statistical analyses; however, we tracked the identities of the individuals to ensure their data selections were honored during the study and to know who completed the survey so that we could compensate them with a gift certificate. The institutional review boards of the University of California, San Diego and the University of California, Irvine approved this study. This study followed the American Association for Public Opinion Research (AAPOR) reporting guideline.

Study participants were invited to select preferences of sharing their data and biospecimens for research use. The preferences for data sharing were honored by the institutions during the study period. Each participant also received periodical reports that listed research activities that involved secondary use of their medical records.

The list of data and biospecimens that a participant could choose to share or not share included 59 data and biospecimen items grouped into 18 categories (Box). This taxonomy was developed based on a pilot study^21^ and 5 focus groups of 18 patients who also provided input on how to best present the selection options on a computer screen and on paper.

Box. **List of Date Elements and Categories**

Contact Information

- Name^a^
- Home address^a^
- Email address^a^
- Telephone number^a^

Demographics

- Age
- Sex
- Race
- Ethnicity
- Sexual orientation^a^

Socioeconomic Status

- Educational level
- Marital status
- Insurance status
- Occupation
- Income

Living Environment and Lifestyle

- Alcohol consumption status
- Recreational drug use
- Smoking status
- Diet^a^
- Physical activity and exercise level^a^
- Stress level^a^
- Social isolation^a^

Sexual Life
Pregnancy History
Adoption History^a^
Body Measurements
Vital Signs
Allergies^a^
Current or Previous Disease or Condition

- Substance abuse–related disease or condition
- Mental health disease or condition
- Sexual or reproductive disease or condition
- Other

Family Health History

- Substance abuse–related disease or condition
- Mental health disease or condition
- Sexual or reproductive disease or condition
- Other

Laboratory Test Results

- Genetic test
- Sexually transmitted disease test
- Drug screening
- DNA sequencing^a^
- Other

Biospecimen

- Tissue
- Blood
- Urine^a^

Imaging Test^a^

- Radiography^a^
- Magnetic resonance imaging^a^
- Computed tomography^a^
- Other^a^

Therapy or Treatment Procedures

- Mental health related^a^
- Genitourinary or reproductive^a^
- Cosmetic^a^
- Bariatric^a^
- Other^a^

Medications

- Mental health related^a^
- Other^a^

Health Care Encounter

- Medical record number^a^
- Visit dates
- Physician’s name
- Specialty of the hospital or clinic
- Clinic location

^a^Items and categories not previously included in our pilot study.^21^

Eleven data categories that encompassed 50 data items, 6 data categories (sexual life, pregnancy history, adoption history, body measurements, vital signs, and allergies) without detailed data items plus 3 biospecimen items grouped into 1 biospecimen category were available for selection (Box). The simple form contained 18 categories, and the detailed form contained 53 detailed items plus 6 data categories (ie, there were 59 sharable items in this detailed form). When 2 interventions (opting method and form layout) were combined, each participant was randomized using simple randomization to 1 of 4 conditions: (1) opt-in simple (n = 322), (2) opt-in detailed (n = 319), (3) opt-out simple (n = 298), and (4) opt-out detailed (n = 307).

For the 4 groups, participants could indicate sharing preferences that could result in 8 combinations of 3 types of researcher’s affiliations (ie, the institution holding their EHRs and biospecimens, including home institution, nonprofit institution, and for-profit institution): (1) do not share, regardless of affiliation; (2) share with the home institution only; (3) share with nonprofit institutions only; (4) share with for-profit institutions only; (5) share with the home institution and nonprofit institutions; (6) share with the home institution and for-profit institutions; (7) share with nonprofit institutions and for-profit institutions; and (8) share with any researcher, regardless of affiliation.

There was no time limit to complete the sharing preferences, which could be changed over time (preferences submitted as of September 31, 2018, were considered in the analysis). Participants indicated their sharing preferences by selecting an item or category that they wanted to share when they received an opt-in form or unselecting what they did not want to share when they received an opt-out form. For the simple forms, when a category was selected, all items that belonged to that category were selected to compare individual items across groups. Participants could assess information about which study used or did not use their data and modify their future sharing choices at any time. The screenshots of our digital consent system are shown in eFigure 1 and eFigure 2 in the Supplement. Once the intervention period was over, a request to complete a satisfaction survey was submitted to assess participant satisfaction with the study and to obtain information about self-reported sociodemographics. Participants had 3 months to complete this survey. Monthly reminder emails were sent, and participants were compensated with a $10 gift card for the completion of the sharing choice form and a $10 gift card for completing the satisfaction survey. They were not compensated when they made changes to previous selections.

We implemented the Short Assessment of Health Literacy, which is designed to assess health literacy by measuring comprehension of the meaning and relation of 18 sets of keywords.^23^ A participant was deemed to have an adequate level of literacy if at least 15 items or 83.3% were answered correctly; otherwise, literacy was deemed to be inadequate according to the Short Assessment of Health Literacy evaluation criteria.^23^

### Statistical Analysis

The homogeneity of the 4 randomization groups by variable of interest was assessed with the χ^2^ test on baseline characteristics. In a univariate analysis, for each of 59 sharable items, a 2 × 2 table was constructed using shared vs not shared as response to a binarized exposure variable (ie, exposure vs reference). An unadjusted odds ratio (OR) and its 95% CI were calculated. Assessed exposure variables included the elicitation form’s opting method (opt-out vs opt-in), form layout (detailed vs simple), patient’s age (≥60 vs <60 years), self-reported health status (very good or better vs worse than very good), health literacy (adequate vs inadequate), sex (female vs male), household income (≥US$125 000 vs <US$125 000 per year), race (white vs nonwhite), educational level (≥4-year college vs <4-year college), and site (site 2 vs site 1). A logistic regression was applied for the model-based adjusted OR after controlling for exposure variables as covariates. Statistical significance was determined by 95% CI of the OR for each sharing choice variable.

## Results

**Figure 1.**
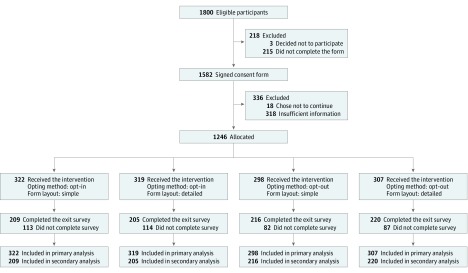
Study Flow Diagram

Of the 1800 patients eligible for this study, 1582 signed a consent form to participate in this study, 1246 (69.2% of eligible participants) who completed their data sharing preference surveys were included in the primary analysis, and 850 (68.2%) of these responded to the exit survey (507 [59.6%] female; 677 [79.6%] white; mean [SD] age, 51.1 [16.7] years). The participant recruitment and randomization processes are summarized in Figure 1. Randomization assignments and characteristics of the participants who completed sharing preferences and who completed the survey are given in Table 1. The higher number in the opt-in group reflects that only this option was available for the 40 participants who elected to use paper forms (simple or detailed). Of 12 variables in Table 1, none had a significant difference among 4 randomized groups.

**Table 1.** Baseline Characteristics by Intervention Group

Characteristic	Patients, No. (%)			
	Opt-In and Simple (n = 322)	Opt-In and Detailed (n = 319)	Opt-Out and Simple (n = 298)	Opt-Out and Detailed (n = 307)
Site				
1	105 (32.6)	99 (31.0)	112 (37.6)	115 (37.5)
2	217 (67.4)	220 (69.0)	186 (62.4)	192 (62.5)
Age, y				
10-19	1 (0.3)	1 (0.3)	0	0
20-29	49 (15.2)	28 (8.8)	41 (13.8)	31 (10.1)
30-39	61 (18.9)	60 (18.8)	58 (19.5)	61 (19.9)
40-49	46 (14.3)	54 (16.9)	41 (13.8)	41 (13.4)
50-59	58 (18.0)	61 (19.1)	51 (17.1)	50 (16.3)
60-69	63 (19.6)	69 (21.6)	66 (22.1)	78 (25.4)
70-79	35 (10.9)	39 (12.2)	36 (12.1)	34 (11.1)
80-89	8 (2.5)	7 (2.0)	4 (1.3)	12 (3.9)
≥90	1 (0.3)	0	1 (0.3)	0
Self-reported health status				
Excellent	31 (9.6)	27 (8.5)	31 (10.4)	36 (11.7)
Very good	103 (32.0)	90 (28.2)	110 (36.9)	93 (30.3)
Good	102 (31.7)	100 (31.3)	103 (34.6)	121 (39.4)
Fair	53 (16.5)	61 (19.1)	46 (15.4)	51 (16.6)
Poor	16 (5.0)	18 (5.6)	8 (2.7)	6 (2.0)
Missing	17 (5.3)	23 (7.2)	0	0
SAHL result				
Adequate	271 (84.2)	272 (85.3)	267 (89.6)	285 (92.8)
Inadequate	34 (10.6)	24 (7.5)	31 (10.4)	22 (7.2)
Missing	17 (4.3)	23 (7.2)	0	0
Sex				
Male	96 (29.8)	78 (24.5)	89 (29.9)	78 (25.4)
Female	113 (35.1)	127 (39.8)	126 (42.3)	141 (45.9)
Other	0	0	1 (0.3)	1 (0.3)
Nonresponse in survey	113 (35.1)	114 (35.7)	82 (27.5)	87 (28.3)
Race				
American Indian/Pacific Islander	3 (0.9)	2 (0.6)	2 (0.7)	2 (0.7)
Asian	21 (6.5)	13 (4.1)	10 (3.4)	17 (5.5)
Black	6 (1.9)	7 (2.2)	7 (2.3)	4 (1.3)
White	157 (48.8)	165 (51.7)	181 (60.7)	174 (56.7)
Multirace or other	22 (6.8)	18 (5.6)	16 (5.4)	23 (7.5)
Nonresponse in survey	113 (35.1)	114 (35.7)	82 (27.5)	87 (28.3)
Ethnicity				
Hispanic	24 (7.5)	25 (7.8)	17 (5.7)	26 (8.5)
Non-Hispanic	185 (57.5)	180 (56.4)	199 (66.8)	194 (63.2)
Nonresponse in survey	113 (35.1)	114 (35.7)	82 (27.5)	87 (28.3)
Educational level				
High school or less	16 (5.0)	9 (2.8)	8 (2.7)	11 (3.6)
More than high school or <4-y college	38 (11.8)	62 (19.4)	47 (15.8)	56 (18.2)
4-y College	75 (23.3)	50 (15.7)	64 (21.5)	64 (2)
Graduate school	80 (24.8)	84 (26.3)	97 (32.6)	89 (29.0)
Nonresponse in survey	113 (35.1)	114 (35.7)	82 (27.5)	87 (28.3)
Household income, $				
<25 000	44 (13.7)	29 (9.1)	35 (11.7)	36 (11.7)
25 000-74 999	40 (12.4)	68 (21.3)	55 (18.5)	58 (18.9)
75 000-124 999	45 (14.0)	43 (13.5)	54 (18.1)	52 (16.9)
125 000-199 999	51 (15.8)	43 (13.5)	42 (14.1)	50 (16.3)
≥200 000	29 (9.0)	22 (6.9)	30 (10.1)	24 (7.8)
Nonresponse in survey	113 (35.1)	114 (35.7)	82 (27.5)	87 (28.3)

Abbreviation: SAHL, Short Assessment of Health Literacy.

A total of 46 participants (3.7%) declined sharing with the home institution, 352 (28.3%) with nonprofit institutions, and 590 (47.4%) with for-profit institutions. A total of 291 patients (23.4%) were willing to share all items with any researcher, whereas 46 (3.7%) were not willing to share any items. The remaining 909 (72.9%) were willing to share selectively, meaning that they wanted to share at least 1 item with at least 1 type of institution with a general preference toward sharing within the institution in which the patient received care, followed by sharing with researchers from nonprofit institutions. A total of 836 patients (67.1%) were willing to share all items with researchers from the home institution.

The analyses based on affiliations focused on those who were not willing to share their data and biospecimens, those who would share their data and biospecimens with the home institution, those who would share their data and biospecimens with the home institution and nonprofit institutions, and those who would share their data and biospecimens with any researcher because the other combinations appeared rarely (4.4%).

Table 2 gives the data sharing preferences of all participants. Demographics, allergies, vital signs, and body measurements were among the items that the participants were most willing to share. Contact information, sexual history, adoption and pregnancy history, and income were the items that the participants were least willing to share.

**Table 2.** Overall Willingness to Share With Different Institutions

Variable	No. (%) of Patients (N = 1246)			
	Sharing With None	Sharing With HI	Sharing With HI and NP	Sharing With HI, NP, and FP
Demographics				
Age	78 (6.3)	321 (25.8)	259 (20.8)	572 (45.9)
Sex	81 (6.5)	317 (25.4)	257 (20.6)	575 (46.1)
Race	91 (7.3)	318 (25.5)	256 (20.5)	564 (45.3)
Ethnicity	94 (7.5)	317 (25.4)	259 (20.8)	560 (44.9)
Sexual orientation	104 (8.3)	315 (25.3)	255 (20.5)	557 (44.7)
Imaging test				
Radiography	97 (7.8)	381 (30.6)	243 (19.5)	514 (41.3)
CT	99 (7.9)	381 (30.6)	242 (19.4)	512 (41.1)
MRI	101 (8.1)	380 (30.5)	242 (19.4)	512 (41.1)
Other	113 (9.1)	377 (30.3)	238 (19.1)	507 (40.7)
Lifestyle				
Exercise	104 (8.3)	352 (28.3)	254 (20.4)	520 (41.7)
Diet	111 (8.9)	346 (27.8)	252 (20.2)	522 (41.9)
Stress	111 (8.9)	351 (28.2)	250 (20.1)	519 (41.7)
Social isolation	117 (9.4)	350 (28.1)	249 (20.0)	515 (41.3)
Smoking	120 (9.6)	342 (27.4)	251 (20.1)	517 (41.5)
Alcohol	121 (9.6)	345 (27.7)	254 (20.4)	512 (41.1)
Drug	126 (10.1)	346 (27.8)	253 (20.3)	507 (40.7)
SES				
Marital status	107 (8.6)	331 (26.6)	256 (20.5)	534 (42.9)
Educational level	114 (9.1)	324 (26.0)	255 (20.5)	535 (42.9)
Occupation	115 (9.2)	334 (26.8)	253 (20.3)	526 (42.2)
Insurance status	124 (10.0)	346 (27.8)	250 (20.1)	510 (40.9)
Income	163 (13.1)	318 (25.5)	247 (19.8)	497 (39.9)
Laboratory test results				
Genetic	113 (9.1)	384 (30.8)	245 (19.7)	492 (39.5)
STD	114 (9.1)	394 (31.6)	235 (18.9)	490 (39.3)
DNA sequencing	114 (9.1)	390 (31.3)	242 (19.4)	488 (39.2)
Drug screening	115 (9.2)	387 (31.1)	240 (19.3)	491 (39.4)
Other	118 (9.5)	392 (31.5)	235 (18.9)	488 (39.2)
Medication				
Other	113 (9.1)	374 (30.0)	239 (19.2)	507 (40.7)
Mental health	122 (9.8)	374 (30.0)	237 (19.0)	499 (40.0)
Disease condition				
Sexual reproductive	114 (9.1)	370 (29.7)	247 (19.8)	501 (40.2)
Mental health	119 (9.6)	363 (29.1)	248 (19.9)	501 (40.2)
Substance abuse	120 (9.6)	365 (29.3)	248 (19.9)	500 (40.1)
Other	125 (10.0)	362 (29.1)	244 (19.6)	501 (40.2)
Biospecimen				
Blood	118 (9.5)	388 (31.1)	231 (18.5)	495 (39.7)
Urine	122 (9.8)	385 (30.9)	231 (18.5)	494 (39.6)
Tissue	126 (10.1)	383 (30.7)	231 (18.5)	492 (39.5)
Family health history				
Mental health	119 (19.6)	380 (30.5)	242 (19.4)	492 (39.5)
Substance abuse	124 (10.0)	379 (30.4)	238 (19.9)	493 (39.6)
Sexual reproductive	124 (10.0)	380 (30.5)	240 (19.3)	490 (39.3)
Other	126 (10.1)	378 (30.3)	239 (19.3)	489 (39.2)
Encounter				
Clinic specialty	119 (9.6)	393 (31.5)	230 (18.5)	489 (39.2)
Clinic location	122 (9.8)	401 (32.2)	226 (18.1)	482 (38.7)
Visit date	125 (10.0)	401 (32.2)	231 (18.5)	474 (38.0)
Physician name	126 (10.1)	401 (32.2)	229 (18.4)	476 (38.2)
MRN	134 (10.8)	413 (33.1)	228 (18.3)	456 (36.6)
Treatment procedure				
Genitourinary/reproductive	126 (10.1)	368 (29.5)	244 (19.6)	495 (39.7)
Mental health	129 (10.4)	371 (29.8)	243 (19.5)	491 (39.4)
Bariatric	129 (10.4)	364 (29.2)	243 (19.5)	498 (40.0)
Cosmetic	131 (10.5)	366 (29.4)	240 (19.3)	496 (39.8)
Other	131 (10.5)	366 (29.4)	244 (19.6)	494 (39.6)
Contact information				
Name	166 (13.3)	467 (37.5)	193 (15.5)	410 (32.9)
Email	182 (14.6)	462 (37.1)	192 (15.4)	402 (32.3)
Home address	189 (15.2)	484 (38.8)	186 (14.9)	375 (30.1)
Telephone	193 (15.5)	485 (38.9)	187 (15.0)	373 (29.9)
Vital signs	89 (7.1)	365 (29.3)	246 (19.7)	534 (42.9)
Allergies	90 (7.2)	353 (28.3)	250 (20.1)	539 (43.3)
Body measurements	108 (8.7)	363 (29.1)	236 (18.9)	526 (42.2)
Pregnancy history	182 (14.6)	368 (29.5)	203 (16.3)	477 (38.3)
Sexual life	194 (15.6)	375 (30.1)	204 (16.4)	462 (37.1)
Adoption history	195 (15.7)	354 (28.4)	201 (16.4)	481 (38.6)

Abbreviations: CT, computed tomography; FP, for profit; HI, home institution; MRI, magnetic resonance imaging; MRN, medical record number; NP, nonprofit; SES, socioeconomic status; STD, sexually transmitted disease.

**Figure 2.**
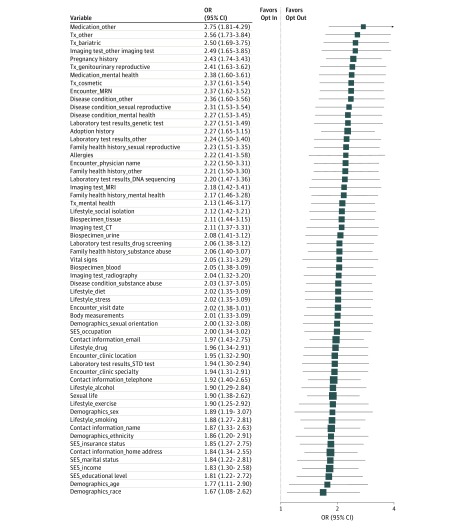
Forest Plot of Unadjusted Odds Ratios (ORs) for Opting Method (Opt-Out vs Opt-In) A total of 59 sharable items or categories were sorted by ORs and shown with their 95% CIs. For each sharable variable, a 2 × 2 table was constructed using a binary outcome (shared vs not shared) and a binary exposure variable (opting method [opt-out vs opt-in]). CT indicates computed tomography; MRI, magnetic resonance imaging; MRN, medical record number; SES, socioeconomic status; STD, sexually transmitted disease; and Tx, treatment.

The sharing preferences were associated with the form’s opting method (opt-out vs opt-in) but not with the layout (detailed vs simple). Participants were willing to share fewer items when they used the opt-in form (Figure 2). Differences according to opting method were significant for all 59 variables (100%). For form layout (eFigure 3 in the Supplement), however, only 14 variables (23.7%) had a significant association with sharing choices. Age of 60 years or older was associated with sharing selections for 56 variables (95%), and adequate health literacy was associated with sharing selections for all 59 variables (100%) (eFigure 4 and eFigure 5 in the Supplement). The associations of opting method with sharing decision remained significant with 1 exception (race) but decreased in magnitude (eFigure 6 in the Supplement) after adjusting for participants’ characteristics and the form layout (from an OR of 1.67 [95% CI, 1.08-2.62] to 1.53 [95% CI, 0.97-2.42]). The adjusted ORs of sharing compared with no sharing for the 59 variables were controlled for form layout, age, educational level, sex, health literacy, household income, self-reported health status, and site in a logistic regression model. For form layout, the number of variables that had a significant association with sharing decision decreased from 14 to 9 after adjusting for participants’ characteristics and the opting method (eFigure 7 in the Supplement).

Participants older than 60 years or deemed to have an adequate health literacy level were more willing to share more items than were their counterparts (eFigure 4 and eFigure 5 in the Supplement). The ORs for all items were greater than 1 and statistically significant, except for sexual life (OR, 1.39; 95% CI, 1.00-1.95), adoption history (OR, 1.37; 95% CI, 0.99-1.92), and pregnancy history (OR, 1.37; 95% CI, 0.98-1.93). Household income, education level, sex, perceived health status, race, and site were not associated with a higher level of sharing for most variables (eFigures 8-13 in the Supplement).

A total of 850 participants (68.2%) completed the satisfaction survey. Of these, 815 (95.9%), could understand the data or information presented in the forms, whereas 17 (2.0%) thought that the choices in the forms were inadequate. A total of 837 (98.4%) enjoyed participating in the study. A total of 517 (60.8%) indicated that having a detailed form layout to make selections had no influence on their sharing decisions, 288 (33.9%) indicated that it made them more willing to share, and 27 (3.2%) indicated that it made them less willing to share their data and biospecimens. The remaining 18 (2.1%) did not answer this question. Consistent with previous findings,^21^ 637 respondents (74.9%) were highly interested in knowing who would use the data or biospecimens, and 683 (80.3%) were also equally willing to share their data and biospecimens for research and health care.

## Discussion

The finding in this study that most patients were willing to share data from their EHRs and biospecimens with researchers is reassuring. Not only can biomedical research benefit from these resources but also a multisite learning health care system^5,24^ can continuously advance as a result of data-driven improvements to processes and associated outcomes. The finding that 955 participants (76.6%) made sharing choices to select at least 1 item that they did not want to share with a particular type of researcher is important when considering that this item might lead to a decision to decline sharing of the whole record if only an all-or-nothing option is available. This finding is important because the item to withhold may not be of relevance to a certain study, but the current all-or-nothing option, if chosen, would remove that patient’s data from all research studies. The finding that 291 participants (23.4%) were willing to share all items with everyone can help plan for studies based on EHRs and biospecimens that are expected to be shared with a broad range of researchers. The finding that only a few participants (46 [3.7%]) were not willing to share any item is also reassuring. Opt-in forms appear to be the most conservative opting method to obtain sharing preferences, resulting in less sharing.

An important finding of this study is that most participants indicated at least 1 item that should not be shared. There was a preference to share the data and biospecimens within the institution in which the patient received care, followed by nonprofit institutions. In a system in which people can choose where to receive care, it seems plausible that a patient selects to receive care in the most trusted institution, and this trust may more easily transfer to the care of data and biospecimens.

The reluctance to share data and biospecimens with researchers from for-profit institutions needs further investigation because the category aggregates highly different industries and further refinement might reveal subgroups that have higher association with declining to share than others. Strategies to convey how data and biospecimens are being used or will be used for research that includes the development of commercial products to improve health outcomes need to be developed and implemented so that patients can provide consent that is truly informed.

In addition, studies that require permission to use the whole EHR for research may consider provisions for participants to decline sharing of specific items and for participants to specify the types of researchers who should be authorized to work with their data. This approach may increase participation and satisfaction.

### Limitations

This study has some limitations. Patients who elect to receive care at academic medical centers may be more familiar with research and more willing to share their data and biospecimens for research than patients who receive care in other types of institutions. In addition, health literacy in general was relatively high in our sample, so the results may be optimistic. However, this optimistic figure may be counterbalanced by the fact that patients who participated in our study may be more concerned about data and biospecimen sharing than those who declined participation. Thus, our recruitment may have selected for individuals who would in general be more concerned about the privacy of their data and biospecimens and tended to remove more items than would those who did not want to participate in this study. There could also be geographic factors: both institutions were located in California, where privacy protections for EHRs are higher than in many other states and in which biomedical and data science research are prominent. However, these limitations do not detract from the findings that it was practical to implement a system that used patient data and biospecimen sharing preferences to guide services that make these resources available for research and that most patients were willing to share their EHR data and biospecimens for research.

## Conclusions

We found that a tiered-permission system that allowed for specific removal of data items or categories of data could be implemented in practice and that it mattered to participants with whom the EHR data and biospecimens would be shared because there were differences in sharing preferences according to the researchers’ affiliations. Participants appreciated being asked about their data and biospecimen sharing preferences. We also found that the way in which sharing preferences were elicited mattered to patients. In this study, data and biospecimen sharing preferences were equivalent across institutions but were different according to the opting method (an opt-out version was associated with more sharing than an opt-in version). A simple form layout that displays data categories was associated with sharing preferences that were equivalent to those elicited from a detailed form layout that displays specific data items.

## Notes

Supplement. **eFigure 1.** Screenshot of participant sharing selection form (opt-in and simple form layout). **eFigure 2.** Screenshot of participant sharing selection form (opt-in and detailed form layout). **eFigure 3.** Forest plot of unadjusted odds ratio for form layout (detailed vs simple). **eFigure 4.** Forest plot of unadjusted odds ratio for age (>=60 vs <60). **eFigure 5.** Forest plot of unadjusted odds ratio for health literacy (adequate vs inadequate). **eFigure 6.** Forest plot of adjusted odds ratio for opting method (opt-out vs opt-in). **eFigure 7.** Forest plot of adjusted odds ratio for form layout (detailed vs simple). **eFigure 8.** Forest plot of unadjusted odds ratio for income (>=$125K vs <$125K). **eFigure 9.** Forest plot of unadjusted odds ratio for education (>=4-year college vs <4-year college). **eFigure 10.** Forest plot of unadjusted odds ratio for gender (female vs male). **eFigure 11.** Forest plot of unadjusted odds ratio for self-reported health (>= very good vs < very good). **eFigure 12.** Forest plot of unadjusted odds ratio for race (white vs nonwhite). **eFigure 13.** Forest plot of unadjusted odds ratio for site (#2 vs #1).
