# Supplementary material for: Patient Perspectives About Decisions to Share Medical Data and Biospecimens for Research
Source: JAMA Netw Open. 2019 Aug 21;2(8):e199550. doi: 10.1001/jamanetworkopen.2019.9550 (PMC6707015; doi:10.1001/jamanetworkopen.2019.9550)
Supplement: Supplement. — eFigure 1. Screenshot of participant sharing selection form (opt-in and simple form layout). eFigure 2. Screenshot of participant sharing selection form (opt-in and detailed form layout). eFigure 3. Forest plot of unadjusted odds ratio for form layout (detailed vs simple). eFigure 4. Forest plot of unadjusted odds ratio for age (>=60 vs <60). eFigure 5. Forest plot of unadjusted odds ratio for health literacy (adequate vs inadequate). eFigure 6. Forest plot of adjusted odds ratio for opting method (opt-out vs opt-in). eFigure 7. Forest plot of adjusted odds ratio for form layout (detailed vs simple). eFigure 8. Forest plot of unadjusted odds ratio for income (>=$125K vs <$125K). eFigure 9. Forest plot of unadjusted odds ratio for education (>=4-year college vs <4-year college). eFigure 10. Forest plot of unadjusted odds ratio for gender (female vs male). eFigure 11. Forest plot of unadjusted odds ratio for self-reported health (>= very good vs < very good). eFigure 12. Forest plot of unadjusted odds ratio for race (white vs nonwhite). eFigure 13. Forest plot of unadjusted odds ratio for site (#2 vs #1). [file jamanetwopen-2-e199550-s001.pdf]

Supplementary Online Content

Kim J, Kim H, Bell E, et al. Patient Perspectives About Decisions to Share Medical Data and Biospecimens for Research. *JAMA Netw Open*. 2019;2(8):e199550. doi:10.1001/jamanetworkopen.2019.9550

eFigure 1. Screenshot of participant sharing selection form (opt-in and simple form layout).

eFigure 2. Screenshot of participant sharing selection form (opt-in and detailed form layout).

eFigure 3. Forest plot of unadjusted odds ratio for form layout (detailed vs. simple).

eFigure 4. Forest plot of unadjusted odds ratio for age (>=60 vs. <60).

eFigure 5. Forest plot of unadjusted odds ratio for health literacy (adequate vs. inadequate).

eFigure 6. Forest plot of adjusted odds ratio for opting method (opt-out vs. opt-in).

eFigure 7. Forest plot of adjusted odds ratio for form layout (detailed vs. simple).

eFigure 8. Forest plot of unadjusted odds ratio for income (>=$125K vs. <$125K).

eFigure 9. Forest plot of unadjusted odds ratio for education (>=4-year-college vs. <4-year-college).

eFigure 10. Forest plot of unadjusted odds ratio for gender (female vs. male).

eFigure 11. Forest plot of unadjusted odds ratio for self-reported health (>= very good vs. <very good).

eFigure 12. Forest plot of unadjusted odds ratio for race (white vs. non-white).

eFigure 13. Forest plot of unadjusted odds ratio for site (#2 vs. #1).

This supplementary material has been provided by the authors to give readers additional information about their work.

© 2019 Kim J et al. *JAMA Network Open.*

eFigure 1. Screenshot of participant sharing selection form (opt-in and simple form layout).

My Sharing Choices

Click here for more information

Please check the data items that you want to share with researchers

What clinical data am I sharing?	Who can access the clinical data I share?		
	My Health System	Non-profit Organizations	For-profit Organizations
	☑ (Check all)	☐ (Check all)	☐ (Check all)
Contact Information	☑	☐	☐
Demographics	☑	☑	☐
Socioeconomic Information	☑	☑	☐
Living Environment and Life Style	☑	☑	☐
Sexual Life	☑	☐	☐
Pregnancy History	☑	☐	☐
Adoption History	☑	☐	☐
Body Measurement	☑	☑	☐
Vital Signs	☑	☑	☐
Allergies	☑	☑	☐
Current or Previous Disease or Condition	☑	☑	☐
Family Health History	☑	☑	☐
Laboratory Test Results	☑	☐	☐
Biospecimen	☑	☑	☐
Imaging Test	☑	☑	☐
Therapy or Treatment Procedures	☑	☑	☐
Medications	☑	☑	☐
Health Care Encounter	☑	☐	☐

© 2019 Kim J et al. *JAMA Network Open.*

eFigure 2. Screenshot of participant sharing selection form (opt-in and detailed form layout).

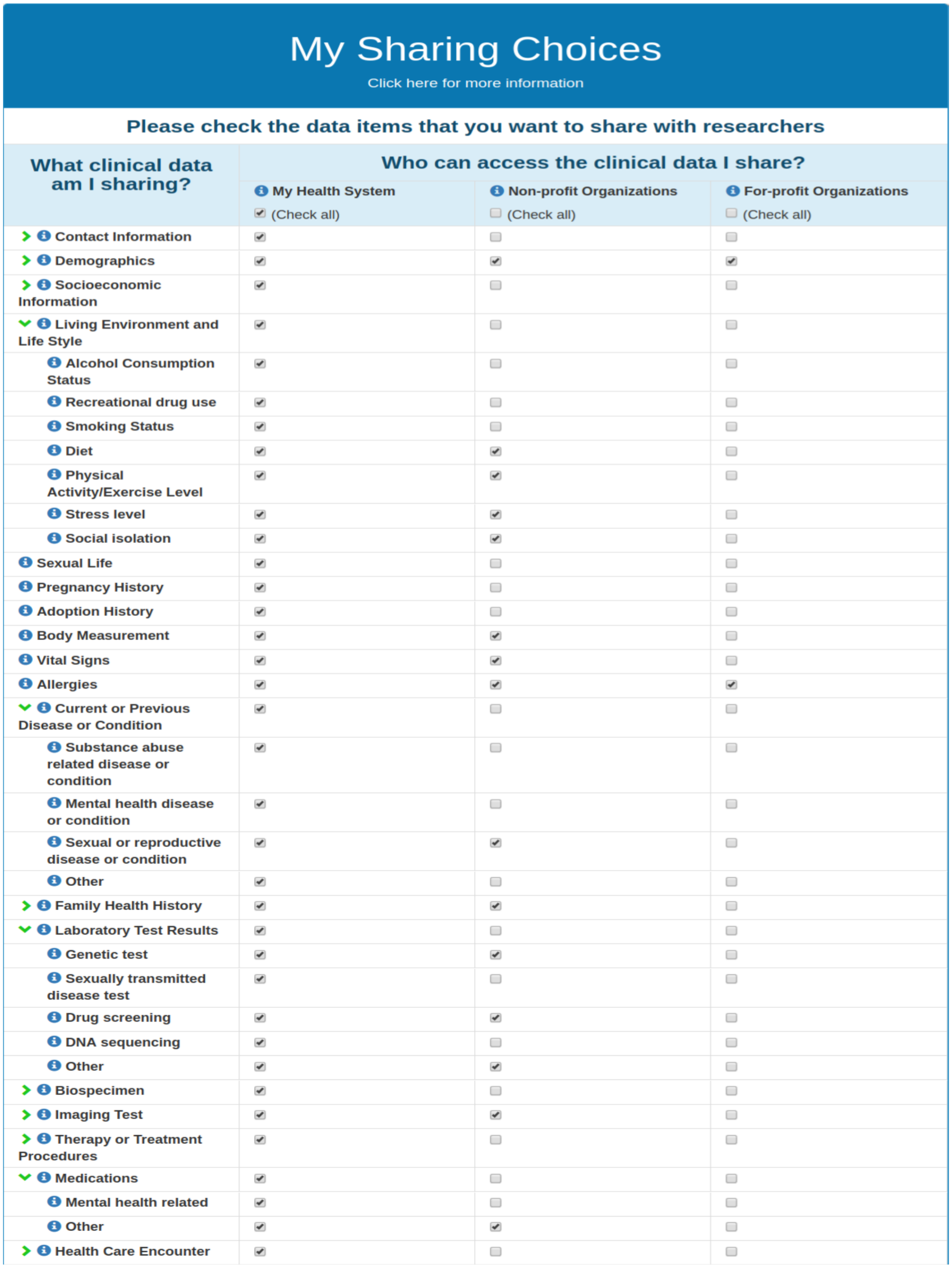

My Sharing Choices

Click here for more information

Please check the data items that you want to share with researchers

What clinical data am I sharing?	Who can access the clinical data I share?		
	My Health System	Non-profit Organizations	For-profit Organizations
	☑ (Check all)	☐ (Check all)	☐ (Check all)
Contact Information	☑	☐	☐
Demographics	☑	☑	☑
Socioeconomic Information	☑	☐	☐
Living Environment and Life Style	☑	☐	☐
Alcohol Consumption Status	☑	☐	☐
Recreational drug use	☑	☐	☐
Smoking Status	☑	☐	☐
Diet	☑	☑	☐
Physical Activity/Exercise Level	☑	☑	☐
Stress level	☑	☑	☐
Social isolation	☑	☑	☐
Sexual Life	☑	☐	☐
Pregnancy History	☑	☐	☐
Adoption History	☑	☐	☐
Body Measurement	☑	☑	☐
Vital Signs	☑	☑	☐
Allergies	☑	☑	☑
Current or Previous Disease or Condition	☑	☐	☐
Substance abuse related disease or condition	☑	☐	☐
Mental health disease or condition	☑	☐	☐
Sexual or reproductive disease or condition	☑	☑	☐
Other	☑	☐	☐
Family Health History	☑	☑	☐
Laboratory Test Results	☑	☐	☐
Genetic test	☑	☑	☐
Sexually transmitted disease test	☑	☐	☐
Drug screening	☑	☑	☐
DNA sequencing	☑	☐	☐
Other	☑	☑	☐
Biospecimen	☑	☐	☐
Imaging Test	☑	☑	☐
Therapy or Treatment Procedures	☑	☐	☐
Medications	☑	☐	☐
Mental health related	☑	☐	☐
Other	☑	☑	☐
Health Care Encounter	☑	☐	☐

© 2019 Kim J et al. *JAMA Network Open.*

eFigure 3. Forest plot of unadjusted odds ratio for form layout (detailed vs. simple).

Form layout (detailed vs. simple)

Variable	Odds Ratio (95% CI)
Biospecimen_Blood	1.41 (0.96 − 2.08)
ImagingTest_X.ray	1.41 (0.93 − 2.16)
ImagingTest_CTscan	1.34 (0.89 − 2.04)
Demographics_Age	1.32 (0.83 − 2.11)
Biospecimen_Urine	1.30 (0.89 − 1.90)
Lifestyle_Exercise	1.29 (0.86 − 1.94)
ImagingTest_MRI	1.28 (0.85 − 1.94)
SES_MaritalStatus	1.26 (0.85 − 1.89)
Demographics_Sex	1.21 (0.77 − 1.91)
Biospecimen_Tissue	1.20 (0.83 − 1.74)
SexualLife	1.16 (0.85 − 1.58)
PregnancyHistory	1.12 (0.82 − 1.54)
Lifestyle_Diet	1.11 (0.75 − 1.64)
Lifestyle_Stress	1.11 (0.75 − 1.64)
SES_Education	1.09 (0.74 − 1.60)
AdoptionHistory	1.07 (0.79 − 1.45)
SES_Occupation	1.07 (0.73 − 1.57)
ImagingTest_OtherImagingTest	0.99 (0.67 − 1.46)
Lifestyle_SocialIsolation	0.99 (0.67 − 1.45)
Encounter_ClinicSpecialty	0.95 (0.65 − 1.39)
Demographics_Race	0.93 (0.61 − 1.43)
Lifestyle_Smoking	0.93 (0.64 − 1.36)
Lifestyle_Alcohol	0.92 (0.63 − 1.34)
Allergies	0.92 (0.60 − 1.41)
SES_InsuranceStatus	0.91 (0.62 − 1.32)
Encounter_ClinicLocation	0.90 (0.62 − 1.31)
Demographics_Ethnicity	0.87 (0.57 − 1.33)
ContactInfo_Name	0.87 (0.63 − 1.21)
BodyMeasurements	0.86 (0.58 − 1.27)
Encounter_VisitDate	0.86 (0.59 − 1.24)
VitalSigns	0.85 (0.55 − 1.31)
Lifestyle_Drug	0.84 (0.58 − 1.22)
Encounter_PhysicianName	0.84 (0.58 − 1.22)
LabTestResults_GeneticTest	0.81 (0.55 − 1.20)
LabTestResults_DNAsequencing	0.80 (0.54 − 1.18)
LabTestResults_STDtest	0.80 (0.54 − 1.17)
LabTestResults_DrugScreening	0.78 (0.53 − 1.15)
Medication_OtherMedication	0.76 (0.51 − 1.11)
LabTestResults_Other	0.74 (0.51 − 1.09)
Encounter_MRN	0.74 (0.52 − 1.07)
TxProcedure_GenitouriaryReproductive	0.73 (0.50 − 1.06)
ContactInfo_Email	0.72 (0.52 − 0.99)
Demographics_SexualOrientation	0.72 (0.48 − 1.08)
DiseaseCondition_SexualReproductive	0.71 (0.48 − 1.05)
TxProcedure_MentalHealth	0.70 (0.48 − 1.01)
TxProcedure_Bariatric	0.70 (0.48 − 1.01)
TxProcedure_Other	0.68 (0.47 − 0.98)
TxProcedure_Cosmetic	0.68 (0.47 − 0.98)
ContactInfo_HomeAddress	0.66 (0.48 − 0.90)
FamilyHealthHistory_MentalHealth	0.66 (0.44 − 0.96)
DiseaseCondition_MentalHealth	0.65 (0.44 − 0.96)
Medication_MentalHealth	0.65 (0.44 − 0.95)
DiseaseCondition_SubstanceAbuse	0.65 (0.44 − 0.94)
ContactInfo_Phone	0.64 (0.47 − 0.87)
FamilyHealthHistory_SubstanceAbuse	0.61 (0.41 − 0.89)
FamilyHealthHistory_SexualReproductive	0.61 (0.41 − 0.89)
DiseaseCondition_Other	0.60 (0.41 − 0.87)
FamilyHealthHistory_Other	0.59 (0.40 − 0.86)
SES_Income	0.52 (0.37 − 0.73)

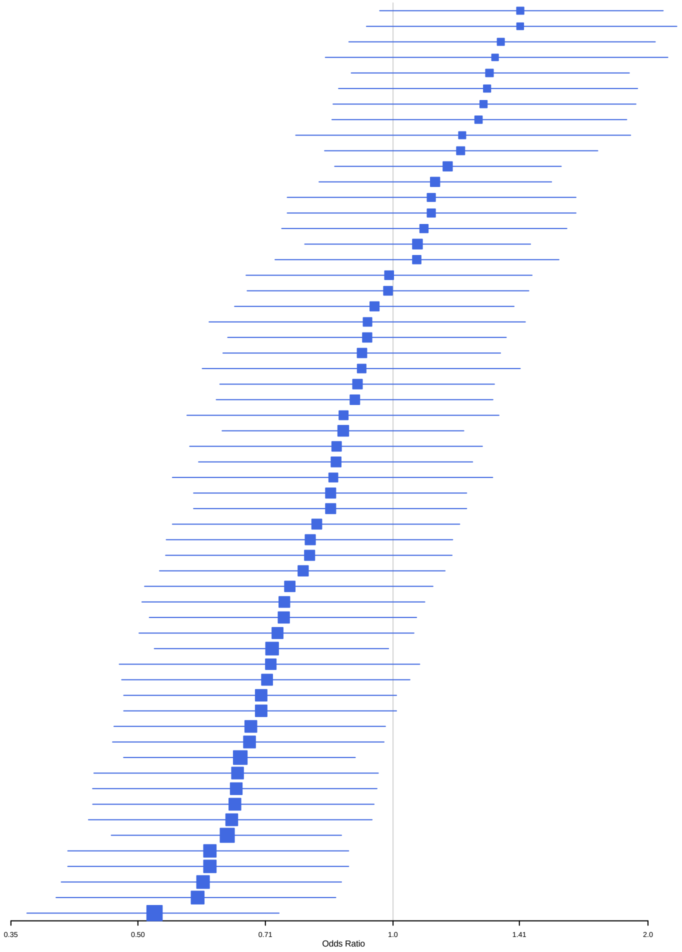

The 59 sharable items were sorted by unadjusted odds ratios and shown with their 95% confidence intervals. For each sharable variable, a 2-by-2 table was constructed using a binary outcome (shared vs. not shared) and a binary exposure variable, form layout (detailed vs. simple). Then the odds ratio and its 95% confidence interval were calculated. Row labels are expressed in the format of “Category_Item”. A category with no item was expressed simply as “Category.” Abbreviations: CT, Computerized Tomography; DNA, DeoxyriboNucleic Acid; MRI, Magnetic Resonance Imaging; MRN, Medical Record Number; SES, Social Economic Status; STD, Sexually Transmitted Disease; TxProcedure, Treatment Procedure.

© 2019 Kim J et al. *JAMA Network Open.*

eFigure 4. Forest plot of unadjusted odds ratio for age (>=60 vs. <60).

Age (>=60 vs. <60)

Variable	Odds Ratio (95% CI)
Biospecimen_Blood	3.70 (2.24 − 6.50)
Allergies	3.58 (2.03 − 6.84)
Biospecimen_Urine	3.38 (2.09 − 5.76)
Lifestyle_Exercise	3.37 (2.00 − 6.04)
Demographics_Age	3.27 (1.81 − 6.45)
ImagingTest_MRI	3.24 (1.92 − 5.81)
VitalSigns	3.21 (1.85 − 6.01)
ImagingTest_CTscan	3.16 (1.87 − 5.67)
Biospecimen_Tissue	3.11 (1.96 − 5.19)
Demographics_Race	3.05 (1.78 − 5.59)
SES_MaritalStatus	3.03 (1.84 − 5.27)
Lifestyle_Stress	2.96 (1.82 − 5.08)
TxProcedure_GenitouriaryReproductive	2.94 (1.86 − 4.85)
Lifestyle_Smoking	2.92 (1.83 − 4.87)
Demographics_SexualOrientation	2.90 (1.76 − 5.05)
Encounter_ClinicSpecialty	2.87 (1.80 − 4.79)
TxProcedure_MentalHealth	2.86 (1.83 − 4.68)
ImagingTest_X.ray	2.85 (1.70 − 5.04)
Demographics_Sex	2.85 (1.63 − 5.35)
Encounter_ClinicLocation	2.80 (1.77 − 4.62)
Lifestyle_Diet	2.77 (1.72 − 4.70)
Lifestyle_Alcohol	2.77 (1.75 − 4.58)
Encounter_PhysicianName	2.76 (1.76 − 4.51)
SES_Occupation	2.75 (1.72 − 4.60)
DiseaseCondition_MentalHealth	2.71 (1.71 − 4.48)
LabTestResults_DNAsequencing	2.70 (1.69 − 4.52)
Encounter_MRN	2.70 (1.75 − 4.33)
Medication_OtherMedication	2.67 (1.67 − 4.47)
Lifestyle_SocialIsolation	2.65 (1.67 − 4.38)
Medication_MentalHealth	2.64 (1.68 − 4.33)
TxProcedure_Other	2.62 (1.69 − 4.21)
Lifestyle_Drug	2.62 (1.68 − 4.24)
Encounter_VisitDate	2.58 (1.65 − 4.19)
DiseaseCondition_SubstanceAbuse	2.58 (1.64 − 4.23)
TxProcedure_Bariatric	2.57 (1.66 − 4.12)
SES_Education	2.55 (1.61 − 4.23)
DiseaseCondition_SexualReproductive	2.55 (1.60 − 4.23)
Demographics_Ethnicity	2.53 (1.53 − 4.43)
ContactInfo_Name	2.53 (1.72 − 3.84)
TxProcedure_Cosmetic	2.49 (1.62 − 3.98)
DiseaseCondition_Other	2.45 (1.58 − 3.95)
LabTestResults_DrugScreening	2.44 (1.55 − 4.01)
ContactInfo_HomeAddress	2.44 (1.69 − 3.58)
FamilyHealthHistory_SubstanceAbuse	2.43 (1.56 − 3.90)
ImagingTest_OtherImagingTest	2.37 (1.50 − 3.90)
FamilyHealthHistory_Other	2.36 (1.53 − 3.77)
ContactInfo_Phone	2.34 (1.63 − 3.41)
SES_InsuranceStatus	2.31 (1.49 − 3.68)
FamilyHealthHistory_SexualReproductive	2.30 (1.49 − 3.68)
FamilyHealthHistory_MentalHealth	2.29 (1.47 − 3.69)
LabTestResults_GeneticTest	2.24 (1.43 − 3.66)
BodyMeasurements	2.21 (1.39 − 3.63)
LabTestResults_STDtest	2.15 (1.38 − 3.48)
LabTestResults_Other	2.14 (1.38 − 3.44)
ContactInfo_Email	2.08 (1.45 − 3.03)
SES_Income	1.58 (1.10 − 2.30)
SexualLife	1.39 (1.00 − 1.95)
AdoptionHistory	1.37 (0.99 − 1.92)
PregnancyHistory	1.37 (0.98 − 1.93)

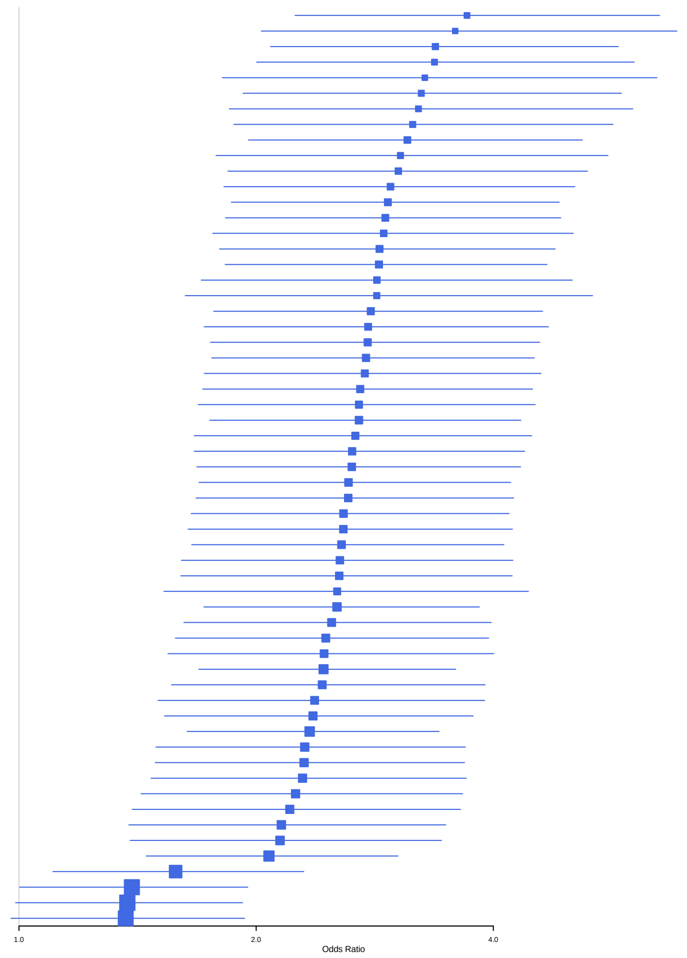

The 59 sharable items were sorted by unadjusted odds ratios and shown with their 95% confidence intervals. For each sharable variable, a 2-by-2 table was constructed using a binary outcome (shared vs. not shared) and a binary exposure variable, age (>= 60 vs. < 60). Then the odds ratio and its 95% confidence interval were calculated. Row labels are expressed in the format of “Category_Item”. A category with no item was expressed simply as “Category.” Abbreviations: CT, Computerized Tomography; DNA, DeoxyriboNucleic Acid; MRI, Magnetic Resonance Imaging; MRN, Medical Record Number; SES, Social Economic Status; STD, Sexually Transmitted Disease; TxProcedure, Treatment Procedure.

© 2019 Kim J et al. *JAMA Network Open.*

eFigure 5. Forest plot of unadjusted odds ratio for health literacy (adequate vs. inadequate).

Health literacy (adequate vs. inadequate)

Variable	Odds Ratio (95% CI)
Demographics_Age	3.87 (2.15 – 6.69)
Demographics_Sex	3.79 (2.11 – 6.56)
ImagingTest_MRI	3.78 (2.21 – 6.28)
ImagingTest_OtherImagingTest	3.76 (2.22 – 6.20)
ImagingTest_X.ray	3.74 (2.16 – 6.26)
Allergies	3.70 (2.09 – 6.31)
ImagingTest_CTscan	3.62 (2.10 – 6.06)
Demographics_Race	3.52 (1.99 – 5.99)
LabTestResults_DrugScreening	3.41 (2.00 – 5.64)
BodyMeasurements	3.37 (1.96 – 5.61)
VitalSigns	3.36 (1.88 – 5.78)
Demographics_Ethnicity	3.31 (1.85 – 5.67)
LabTestResults_Other	3.23 (1.88 – 5.36)
TxProcedure_Cosmetic	3.18 (1.91 – 5.16)
TxProcedure_Bariatric	3.09 (1.84 – 5.05)
Demographics_SexualOrientation	3.07 (1.75 – 5.20)
Medication_OtherMedication	3.05 (1.76 – 5.11)
LabTestResults_GeneticTest	3.05 (1.76 – 5.10)
FamilyHealthHistory_Other	3.04 (1.79 – 5.00)
TxProcedure_Other	3.03 (1.80 – 4.93)
Encounter_ClinicLocation	3.01 (1.79 – 4.91)
DiseaseCondition_Other	3.00 (1.77 – 4.93)
Encounter_VisitDate	2.98 (1.77 – 4.86)
SES_Education	2.94 (1.71 – 4.86)
LabTestResults_DNAsequencing	2.93 (1.69 – 4.89)
Encounter_ClinicSpecialty	2.92 (1.72 – 4.80)
DiseaseCondition_SubstanceAbuse	2.91 (1.70 – 4.82)
DiseaseCondition_MentalHealth	2.90 (1.70 – 4.81)
DiseaseCondition_SexualReproductive	2.89 (1.67 – 4.82)
FamilyHealthHistory_SubstanceAbuse	2.88 (1.68 – 4.76)
FamilyHealthHistory_MentalHealth	2.78 (1.61 – 4.63)
TxProcedure_GenitouriaryReproductive	2.77 (1.62 – 4.57)
Biospecimen_Blood	2.77 (1.62 – 4.57)
LabTestResults_STDtest	2.72 (1.56 – 4.57)
Lifestyle_Diet	2.72 (1.55 – 4.57)
Lifestyle_Stress	2.72 (1.55 – 4.57)
Biospecimen_Urine	2.71 (1.58 – 4.46)
SES_InsuranceStatus	2.70 (1.58 – 4.46)
Encounter_PhysicianName	2.70 (1.60 – 4.41)
Encounter_MRN	2.69 (1.61 – 4.37)
Lifestyle_Exercise	2.69 (1.52 – 4.57)
FamilyHealthHistory_SexualReproductive	2.68 (1.56 – 4.47)
Biospecimen_Tissue	2.67 (1.57 – 4.41)
Lifestyle_Alcohol	2.64 (1.53 – 4.39)
Lifestyle_Smoking	2.64 (1.53 – 4.39)
Medication_MentalHealth	2.62 (1.52 – 4.35)
Lifestyle_SocialIsolation	2.61 (1.50 – 4.38)
SES_Occupation	2.58 (1.48 – 4.33)
SES_MaritalStatus	2.52 (1.43 – 4.26)
SES_Income	2.50 (1.52 – 4.00)
PregnancyHistory	2.41 (1.48 – 3.82)
TxProcedure_MentalHealth	2.39 (1.39 – 3.95)
Lifestyle_Drug	2.37 (1.36 – 3.97)
ContactInfo_Email	2.34 (1.45 – 3.68)
ContactInfo_Name	2.32 (1.43 – 3.67)
ContactInfo_Phone	2.23 (1.39 – 3.49)
ContactInfo_HomeAddress	2.20 (1.37 – 3.45)
AdoptionHistory	2.20 (1.36 – 3.49)
SexualLife	1.98 (1.21 – 3.14)

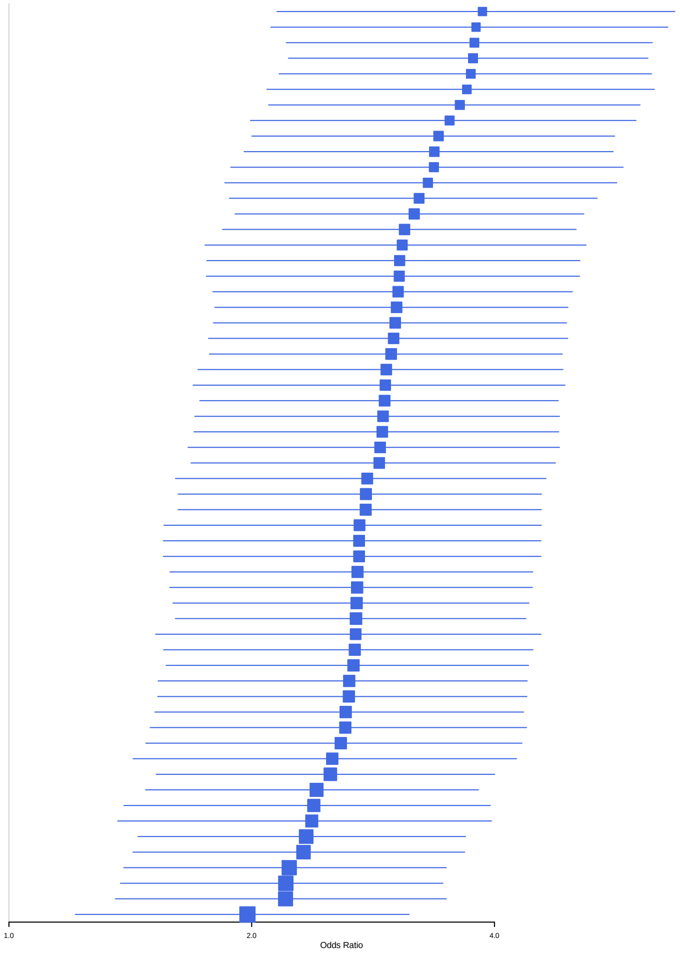

The 59 sharable items were sorted by unadjusted odds ratios and shown with their 95% confidence intervals. For each sharable variable, a 2-by-2 table was constructed using a binary outcome (shared vs. not shared) and a binary exposure variable, health literacy (adequate vs. inadequate). Then the odds ratio and its 95% confidence interval were calculated. Row labels are expressed in the format of “Category_Item”. A category with no item was expressed simply as “Category.” Abbreviations: CT, Computerized Tomography; DNA, DeoxyriboNucleic Acid; MRI, Magnetic Resonance Imaging; MRN, Medical Record Number; SES, Social Economic Status; STD, Sexually Transmitted Disease; TxProcedure, Treatment Procedure.

© 2019 Kim J et al. *JAMA Network Open.*

eFigure 6. Forest plot of adjusted odds ratio for opting method (opt-out vs. opt-in).

Opting method (opt−out vs. opt−in)

Variable	Adjusted Odds Ratio (95% CI)
Medication_OtherMedication	2.36 (1.51 − 3.69)
Encounter_MRN	2.33 (1.56 − 3.47)
TxProcedure_Other	2.23 (1.48 − 3.37)
Medication_MentalHealth	2.22 (1.46 − 3.38)
Encounter_PhysicianName	2.20 (1.47 − 3.29)
TxProcedure_Bariatric	2.16 (1.43 − 3.28)
ImagingTest_OtherImagingTest	2.14 (1.37 − 3.32)
DiseaseCondition_MentalHealth	2.12 (1.39 − 3.23)
TxProcedure_GenitouriaryReproductive	2.11 (1.39 − 3.21)
DiseaseCondition_SexualReproductive	2.11 (1.37 − 3.24)
TxProcedure_Cosmetic	2.09 (1.39 − 3.14)
DiseaseCondition_Other	2.08 (1.37 − 3.16)
ImagingTest_MRI	2.05 (1.31 − 3.22)
Biospecimen_Blood	2.04 (1.34 − 3.09)
Biospecimen_Urine	2.02 (1.34 − 3.05)
TxProcedure_MentalHealth	2.01 (1.34 − 3.01)
Allergies	2.00 (1.24 − 3.23)
PregnancyHistory	1.99 (1.40 − 2.83)
ImagingTest_CTscan	1.99 (1.26 − 3.13)
LabTestResults_DNAsequencing	1.97 (1.28 − 3.03)
LabTestResults_GeneticTest	1.97 (1.27 − 3.04)
Biospecimen_Tissue	1.96 (1.30 − 2.95)
FamilyHealthHistory_SexualReproductive	1.95 (1.29 − 2.96)
FamilyHealthHistory_MentalHealth	1.95 (1.28 − 2.97)
VitalSigns	1.92 (1.20 − 3.08)
ImagingTest_X.ray	1.92 (1.22 − 3.02)
Encounter_VisitDate	1.92 (1.28 − 2.86)
FamilyHealthHistory_Other	1.91 (1.27 − 2.90)
Encounter_ClinicLocation	1.90 (1.27 − 2.84)
ContactInfo_Name	1.90 (1.34 − 2.69)
Encounter_ClinicSpecialty	1.89 (1.26 − 2.84)
Lifestyle_SocialIsolation	1.87 (1.23 − 2.85)
Lifestyle_Stress	1.85 (1.21 − 2.84)
Lifestyle_Diet	1.85 (1.21 − 2.83)
ContactInfo_HomeAddress	1.84 (1.32 − 2.57)
SES_Occupation	1.83 (1.21 − 2.78)
ContactInfo_Phone	1.83 (1.31 − 2.56)
AdoptionHistory	1.81 (1.29 − 2.55)
DiseaseCondition_SubstanceAbuse	1.81 (1.19 − 2.74)
ContactInfo_Email	1.80 (1.28 − 2.54)
LabTestResults_Other	1.80 (1.17 − 2.77)
SES_MaritalStatus	1.80 (1.18 − 2.74)
FamilyHealthHistory_SubstanceAbuse	1.78 (1.18 − 2.69)
Lifestyle_Exercise	1.77 (1.15 − 2.74)
Demographics_SexualOrientation	1.77 (1.14 − 2.75)
LabTestResults_DrugScreening	1.76 (1.14 − 2.69)
Demographics_Sex	1.75 (1.08 − 2.86)
BodyMeasurements	1.74 (1.12 − 2.68)
LabTestResults_STDtest	1.72 (1.13 − 2.63)
Lifestyle_Drug	1.72 (1.15 − 2.58)
Demographics_Age	1.72 (1.05 − 2.80)
Lifestyle_Smoking	1.72 (1.14 − 2.58)
Lifestyle_Alcohol	1.71 (1.14 − 2.57)
SES_Education	1.70 (1.13 − 2.57)
SES_InsuranceStatus	1.69 (1.13 − 2.51)
SES_Income	1.63 (1.14 − 2.33)
SexualLife	1.63 (1.17 − 2.27)
Demographics_Ethnicity	1.58 (1.00 − 2.50)
Demographics_Race	1.53 (0.97 − 2.42)

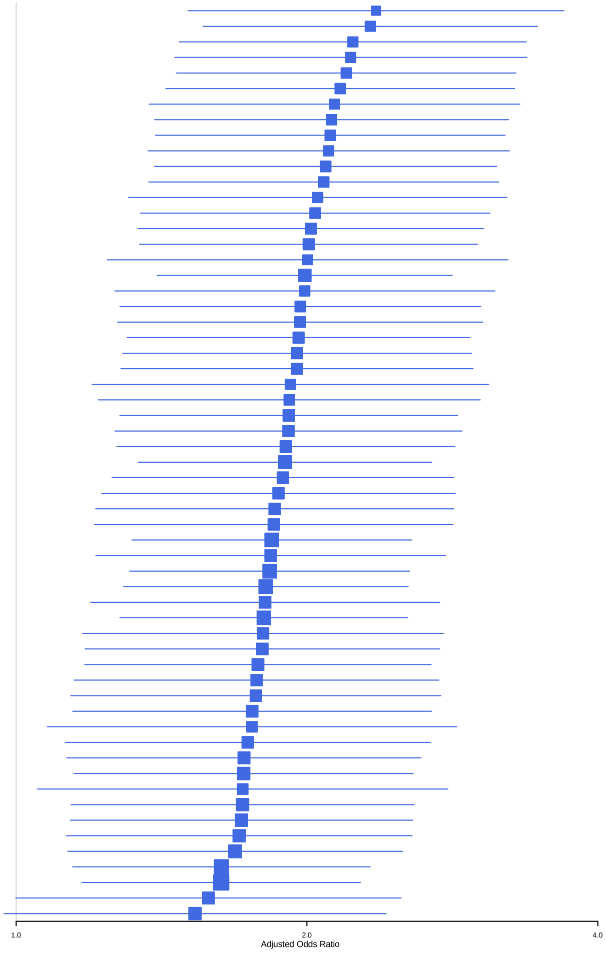

The 59 sharable items were sorted by adjusted odds ratios from the multivariate model (See the Methods for details) and shown with their 95% confidence intervals. Row labels are expressed in the format of “Category_Item”. A category with no item was expressed simply as “Category.” Abbreviations: CT, Computerized Tomography; DNA, DeoxyriboNucleic Acid; MRI, Magnetic Resonance Imaging; MRN, Medical Record Number; SES, Social Economic Status; STD, Sexually Transmitted Disease; TxProcedure, Treatment Procedure.

© 2019 Kim J et al. *JAMA Network Open.*

eFigure 7. Forest plot of adjusted odds ratio for form layout (detailed vs. simple).

Form layout (detailed vs. simple)

Variable	Adjusted Odds Ratio (95% CI)
ImagingTest_X.ray	1.41 (0.90 − 2.20)
Lifestyle_Exercise	1.33 (0.87 − 2.04)
ImagingTest_CTscan	1.32 (0.85 − 2.05)
Biospecimen_Blood	1.31 (0.87 − 1.96)
Demographics_Age	1.31 (0.81 − 2.11)
SexualLife	1.28 (0.92 − 1.78)
Demographics_Sex	1.26 (0.78 − 2.04)
ImagingTest_MRI	1.26 (0.81 − 1.94)
Biospecimen_Urine	1.25 (0.84 − 1.87)
SES_MaritalStatus	1.23 (0.82 − 1.86)
Biospecimen_Tissue	1.23 (0.82 − 1.82)
Lifestyle_Stress	1.18 (0.78 − 1.78)
Lifestyle_Diet	1.17 (0.77 − 1.78)
SES_Occupation	1.13 (0.75 − 1.69)
PregnancyHistory	1.11 (0.79 − 1.57)
ImagingTest_OtherImagingTest	1.10 (0.72 − 1.69)
SES_Education	1.10 (0.74 − 1.65)
AdoptionHistory	1.10 (0.79 − 1.53)
Lifestyle_SocialIsolation	1.08 (0.72 − 1.63)
Lifestyle_Alcohol	0.98 (0.66 − 1.46)
Lifestyle_Smoking	0.97 (0.65 − 1.45)
Demographics_Ethnicity	0.97 (0.62 − 1.52)
SES_InsuranceStatus	0.97 (0.65 − 1.43)
Demographics_Race	0.95 (0.60 − 1.48)
Lifestyle_Drug	0.92 (0.62 − 1.37)
BodyMeasurements	0.92 (0.60 − 1.41)
Encounter_ClinicSpecialty	0.91 (0.61 − 1.36)
Allergies	0.89 (0.56 − 1.40)
Encounter_ClinicLocation	0.86 (0.58 − 1.28)
LabTestResults_GeneticTest	0.86 (0.56 − 1.31)
LabTestResults_Other	0.85 (0.56 − 1.30)
Encounter_VisitDate	0.85 (0.57 − 1.25)
Medication_OtherMedication	0.85 (0.55 − 1.29)
LabTestResults_DrugScreening	0.83 (0.55 − 1.27)
LabTestResults_STDtest	0.82 (0.54 − 1.23)
VitalSigns	0.82 (0.52 − 1.29)
ContactInfo_Name	0.81 (0.58 − 1.15)
LabTestResults_DNAsequencing	0.81 (0.54 − 1.23)
TxProcedure_GenitouriaryReproductive	0.80 (0.54 − 1.20)
Encounter_PhysicianName	0.79 (0.54 − 1.17)
TxProcedure_Bariatric	0.77 (0.52 − 1.15)
Demographics_SexualOrientation	0.76 (0.50 − 1.18)
ContactInfo_Email	0.75 (0.54 − 1.05)
TxProcedure_Other	0.75 (0.50 − 1.11)
DiseaseCondition_SexualReproductive	0.74 (0.49 − 1.12)
TxProcedure_Cosmetic	0.73 (0.49 − 1.09)
Encounter_MRN	0.71 (0.48 − 1.04)
TxProcedure_MentalHealth	0.70 (0.47 − 1.03)
FamilyHealthHistory_MentalHealth	0.68 (0.45 − 1.02)
DiseaseCondition_SubstanceAbuse	0.67 (0.45 − 1.01)
Medication_MentalHealth	0.66 (0.44 − 0.99)
DiseaseCondition_MentalHealth	0.66 (0.44 − 0.99)
FamilyHealthHistory_SexualReproductive	0.65 (0.43 − 0.97)
FamilyHealthHistory_SubstanceAbuse	0.64 (0.43 − 0.97)
DiseaseCondition_Other	0.64 (0.42 − 0.96)
ContactInfo_Phone	0.63 (0.45 − 0.88)
FamilyHealthHistory_Other	0.62 (0.42 − 0.94)
ContactInfo_HomeAddress	0.62 (0.45 − 0.86)
SES_Income	0.56 (0.39 − 0.81)

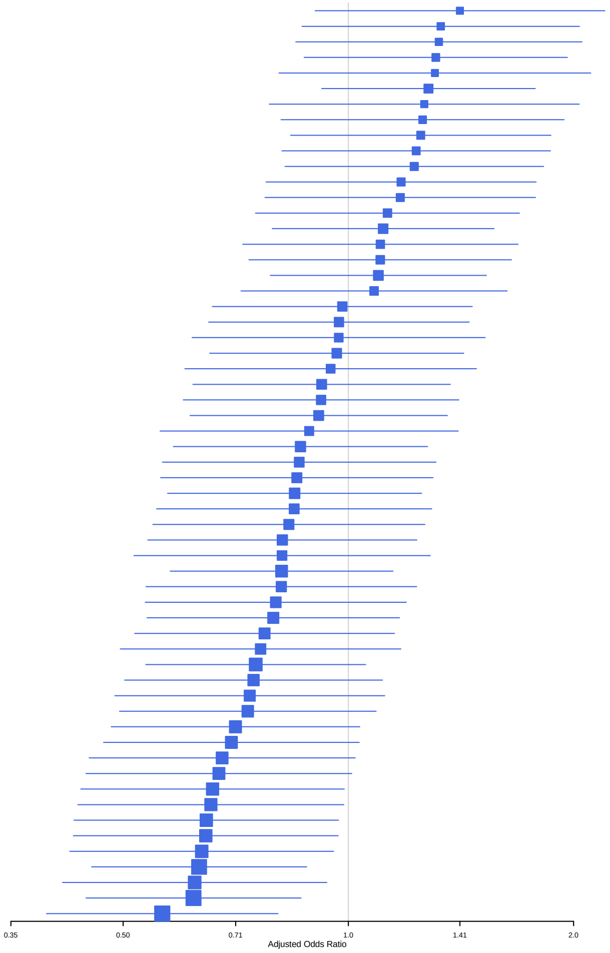

The 59 sharable items were sorted by adjusted odds ratios from the multivariate model (See the Methods for details) and shown with their 95% confidence intervals. Row labels are expressed in the format of “Category_Item”. A category with no item was expressed simply as “Category.” Abbreviations: CT, Computerized Tomography; DNA, DeoxyriboNucleic Acid; MRI, Magnetic Resonance Imaging; MRN, Medical Record Number; SES, Social Economic Status; STD, Sexually Transmitted Disease; TxProcedure, Treatment Procedure.

© 2019 Kim J et al. *JAMA Network Open.*

eFigure 8. Forest plot of unadjusted odds ratio for income (>=$125K vs. <$125K)

Income (>= $125K vs. < $125K)

Variable	Odds Ratio (95% CI)
DiseaseCondition_SubstanceAbuse	2.03 (1.15 − 3.79)
Demographics_SexualOrientation	2.01 (1.08 − 4.05)
TxProcedure_GenitouriaryReproductive	1.88 (1.10 − 3.40)
Demographics_Sex	1.87 (0.91 − 4.25)
TxProcedure_MentalHealth	1.87 (1.10 − 3.32)
DiseaseCondition_Other	1.86 (1.07 − 3.41)
TxProcedure_Bariatric	1.84 (1.07 − 3.33)
FamilyHealthHistory_SubstanceAbuse	1.82 (1.04 − 3.34)
Medication_MentalHealth	1.81 (1.05 − 3.28)
Demographics_Age	1.81 (0.88 − 4.12)
DiseaseCondition_SexualReproductive	1.79 (1.01 − 3.36)
LabTestResults_DrugScreening	1.79 (1.01 − 3.36)
Lifestyle_Alcohol	1.78 (1.03 − 3.22)
AdoptionHistory	1.76 (1.13 − 2.83)
LabTestResults_Other	1.75 (0.98 − 3.29)
LabTestResults_STDtest	1.74 (1.00 − 3.22)
LabTestResults_DNAsequencing	1.74 (1.00 − 3.22)
FamilyHealthHistory_SexualReproductive	1.74 (1.00 − 3.21)
TxProcedure_Cosmetic	1.74 (1.02 − 3.10)
TxProcedure_Other	1.74 (1.02 − 3.09)
Lifestyle_Diet	1.71 (0.96 − 3.23)
Lifestyle_Stress	1.71 (0.96 − 3.23)
BodyMeasurements	1.71 (0.92 − 3.37)
FamilyHealthHistory_Other	1.71 (0.99 − 3.10)
ImagingTest_X.ray	1.71 (0.94 − 3.29)
Lifestyle_Smoking	1.68 (0.97 − 3.05)
Allergies	1.68 (0.86 − 3.53)
DiseaseCondition_MentalHealth	1.67 (0.97 − 3.04)
ImagingTest_OtherImagingTest	1.66 (0.95 − 3.08)
Lifestyle_Drug	1.64 (0.96 − 2.94)
Biospecimen_Tissue	1.64 (0.97 − 2.88)
Biospecimen_Urine	1.61 (0.95 − 2.83)
Lifestyle_Exercise	1.60 (0.89 − 3.03)
ImagingTest_MRI	1.59 (0.89 − 3.00)
ImagingTest_CTscan	1.59 (0.89 − 3.00)
Biospecimen_Blood	1.57 (0.93 − 2.78)
FamilyHealthHistory_MentalHealth	1.57 (0.90 − 2.85)
Medication_OtherMedication	1.56 (0.89 − 2.90)
SES_Occupation	1.54 (0.90 − 2.77)
Lifestyle_SocialIsolation	1.54 (0.89 − 2.81)
LabTestResults_GeneticTest	1.53 (0.88 − 2.79)
SexualLife	1.49 (0.97 − 2.34)
SES_MaritalStatus	1.48 (0.86 − 2.66)
SES_Education	1.45 (0.84 − 2.60)
Demographics_Race	1.42 (0.75 − 2.83)
Demographics_Ethnicity	1.37 (0.73 − 2.75)
VitalSigns	1.36 (0.72 − 2.73)
PregnancyHistory	1.29 (0.83 − 2.03)
SES_InsuranceStatus	1.24 (0.75 − 2.10)
Encounter_ClinicLocation	1.15 (0.70 − 1.94)
Encounter_ClinicSpecialty	1.08 (0.66 − 1.83)
Encounter_MRN	1.08 (0.67 − 1.76)
ContactInfo_Email	1.07 (0.71 − 1.65)
Encounter_VisitDate	1.06 (0.65 − 1.77)
ContactInfo_HomeAddress	1.06 (0.70 − 1.60)
SES_Income	1.05 (0.68 − 1.65)
ContactInfo_Name	1.02 (0.67 − 1.58)
Encounter_PhysicianName	1.01 (0.63 − 1.67)
ContactInfo_Phone	0.99 (0.67 − 1.50)

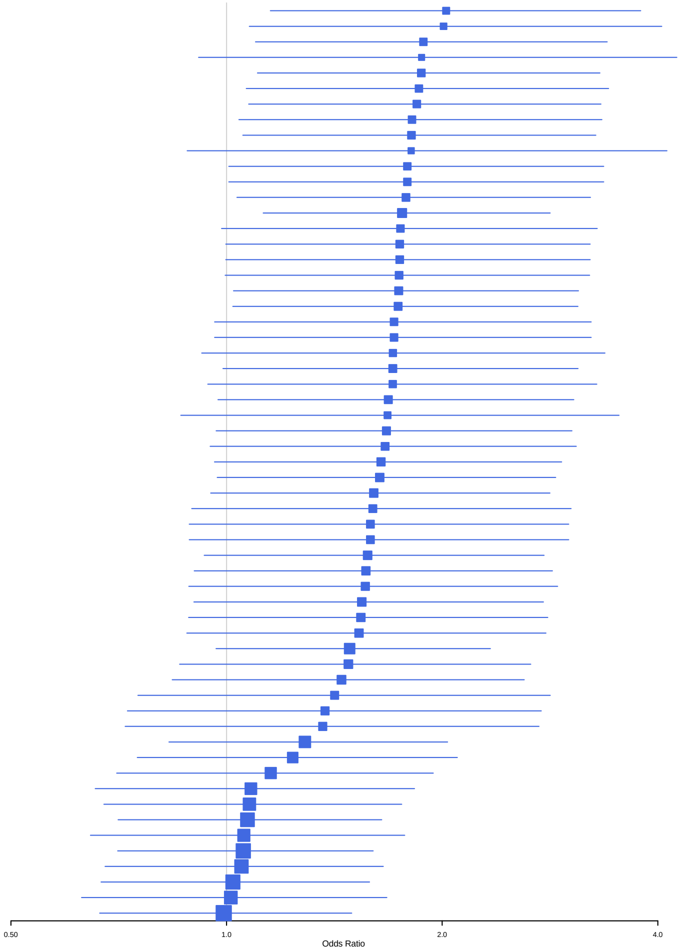

The 59 sharable items were sorted by unadjusted odds ratios and shown with their 95% confidence intervals. For each sharable variable, a 2-by-2 table was constructed using a binary outcome (shared vs. not shared) and a binary exposure variable, income (>=$125K vs. <$125K). Then the odds ratio and its 95% confidence interval were calculated. Row labels are expressed in the format of “Category_Item”. A category with no item was expressed simply as “Category.” Abbreviations: CT, Computerized Tomography; DNA, DeoxyriboNucleic Acid; MRI, Magnetic Resonance Imaging; MRN, Medical Record Number; SES, Social Economic Status; STD, Sexually Transmitted Disease; TxProcedure, Treatment Procedure.

eFigure 9. Forest plot of unadjusted odds ratio for education (>=4-year-college vs. <4-year-college).

Education (>= 4−year−college vs. < 4−year−college)

Variable	Odds Ratio (95% CI)
Demographics_Sex	1.80 (0.93 − 3.40)
SES_Occupation	1.72 (1.03 − 2.83)
VitalSigns	1.71 (0.92 − 3.12)
LabTestResults_DrugScreening	1.71 (1.01 − 2.88)
Demographics_Age	1.69 (0.86 − 3.22)
Allergies	1.62 (0.86 − 2.97)
FamilyHealthHistory_SubstanceAbuse	1.60 (0.96 − 2.65)
Lifestyle_Alcohol	1.58 (0.95 − 2.60)
Lifestyle_Drug	1.58 (0.95 − 2.60)
Lifestyle_Exercise	1.58 (0.90 − 2.70)
BodyMeasurements	1.56 (0.87 − 2.73)
Lifestyle_Diet	1.56 (0.90 − 2.64)
Lifestyle_Stress	1.56 (0.90 − 2.64)
SES_InsuranceStatus	1.54 (0.94 − 2.50)
SES_Education	1.51 (0.89 − 2.53)
Lifestyle_Smoking	1.51 (0.89 − 2.50)
Demographics_SexualOrientation	1.48 (0.83 − 2.57)
SES_MaritalStatus	1.48 (0.87 − 2.46)
FamilyHealthHistory_SexualReproductive	1.47 (0.87 − 2.46)
FamilyHealthHistory_Other	1.47 (0.87 − 2.43)
DiseaseCondition_SubstanceAbuse	1.46 (0.87 − 2.42)
DiseaseCondition_Other	1.46 (0.87 − 2.42)
Lifestyle_SocialIsolation	1.44 (0.84 − 2.43)
LabTestResults_Other	1.42 (0.82 − 2.41)
DiseaseCondition_SexualReproductive	1.37 (0.79 − 2.32)
Demographics_Race	1.37 (0.73 − 2.51)
LabTestResults_DNAsequencing	1.37 (0.80 − 2.29)
TxProcedure_Bariatric	1.34 (0.80 − 2.19)
FamilyHealthHistory_MentalHealth	1.31 (0.76 − 2.20)
TxProcedure_GenitouriaryReproductive	1.30 (0.78 − 2.13)
TxProcedure_Cosmetic	1.30 (0.78 − 2.13)
Demographics_Ethnicity	1.29 (0.67 − 2.38)
LabTestResults_STDtest	1.28 (0.74 − 2.14)
LabTestResults_GeneticTest	1.25 (0.72 − 2.11)
Medication_OtherMedication	1.25 (0.71 − 2.13)
TxProcedure_Other	1.22 (0.73 − 2.01)
DiseaseCondition_MentalHealth	1.22 (0.71 − 2.04)
ImagingTest_X.ray	1.18 (0.65 − 2.06)
ImagingTest_MRI	1.15 (0.64 − 2.00)
ImagingTest_CTscan	1.15 (0.64 − 2.00)
Biospecimen_Blood	1.10 (0.64 − 1.82)
ImagingTest_OtherImagingTest	1.07 (0.61 − 1.84)
Biospecimen_Urine	1.07 (0.63 − 1.78)
PregnancyHistory	1.06 (0.67 − 1.65)
AdoptionHistory	1.06 (0.68 − 1.62)
TxProcedure_MentalHealth	1.06 (0.63 − 1.73)
Biospecimen_Tissue	1.05 (0.62 − 1.74)
Medication_MentalHealth	1.04 (0.60 − 1.73)
SES_Income	1.02 (0.63 − 1.61)
SexualLife	1.02 (0.65 − 1.55)
Encounter_VisitDate	1.00 (0.58 − 1.66)
Encounter_ClinicLocation	0.98 (0.57 − 1.63)
Encounter_ClinicSpecialty	0.97 (0.56 − 1.63)
Encounter_PhysicianName	0.92 (0.54 − 1.52)
Encounter_MRN	0.88 (0.52 − 1.44)
ContactInfo_Email	0.78 (0.49 − 1.22)
ContactInfo_Phone	0.73 (0.46 − 1.13)
ContactInfo_HomeAddress	0.67 (0.42 − 1.04)
ContactInfo_Name	0.64 (0.38 − 1.03)

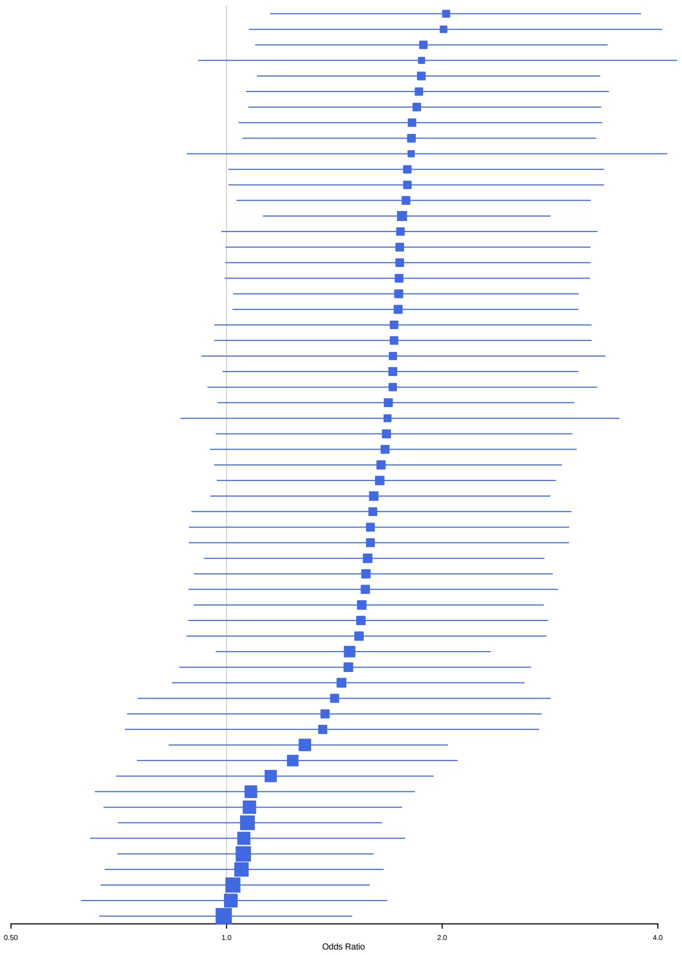

The 59 sharable items were sorted by unadjusted odds ratios and shown with their 95% confidence intervals. For each sharable variable, a 2-by-2 table was constructed using a binary outcome (shared vs. not shared) and a binary exposure variable, education (>= 4-year-college vs. < 4-year-college). Then the odds ratio and its 95% confidence interval were calculated. Row labels are expressed in the format of “Category_Item”. A category with no item was expressed simply as “Category.” Abbreviations: CT, Computerized Tomography; DNA, DeoxyriboNucleic Acid; MRI, Magnetic Resonance Imaging; MRN, Medical Record Number; SES, Social Economic Status; STD, Sexually Transmitted Disease; TxProcedure, Treatment Procedure.

© 2019 Kim J et al. *JAMA Network Open.*

eFigure 10. Forest plot of unadjusted odds ratio for gender (female vs. male).

Gender (female vs. male)

Variable	Odds Ratio (95% CI)
PregnancyHistory	1.64 (1.09 − 2.48)
LabTestResults_DrugScreening	1.13 (0.67 − 1.89)
LabTestResults_Other	1.09 (0.64 − 1.82)
AdoptionHistory	1.06 (0.71 − 1.59)
SES_Occupation	0.99 (0.59 − 1.62)
Lifestyle_Alcohol	0.98 (0.59 − 1.59)
SES_InsuranceStatus	0.94 (0.58 − 1.52)
FamilyHealthHistory_SubstanceAbuse	0.93 (0.55 − 1.53)
Lifestyle_Smoking	0.92 (0.55 − 1.52)
Lifestyle_Drug	0.92 (0.55 − 1.50)
LabTestResults_STDtest	0.91 (0.54 − 1.51)
Lifestyle_Diet	0.91 (0.53 − 1.54)
Lifestyle_Stress	0.91 (0.53 − 1.54)
DiseaseCondition_SubstanceAbuse	0.90 (0.54 − 1.48)
ImagingTest_OtherImagingTest	0.90 (0.53 − 1.50)
Lifestyle_SocialIsolation	0.90 (0.53 − 1.50)
ImagingTest_X.ray	0.88 (0.50 − 1.51)
SES_Education	0.87 (0.51 − 1.45)
DiseaseCondition_SexualReproductive	0.86 (0.50 − 1.44)
ImagingTest_MRI	0.85 (0.49 − 1.46)
ImagingTest_CTscan	0.85 (0.49 − 1.46)
LabTestResults_DNAsequencing	0.85 (0.50 − 1.42)
Lifestyle_Exercise	0.85 (0.49 − 1.46)
SES_MaritalStatus	0.85 (0.50 − 1.41)
FamilyHealthHistory_Other	0.85 (0.50 − 1.39)
LabTestResults_GeneticTest	0.84 (0.49 − 1.40)
VitalSigns	0.84 (0.44 − 1.53)
Biospecimen_Urine	0.80 (0.48 − 1.30)
FamilyHealthHistory_SexualReproductive	0.80 (0.47 − 1.33)
DiseaseCondition_Other	0.79 (0.47 − 1.31)
Encounter_ClinicSpecialty	0.79 (0.47 − 1.29)
Allergies	0.78 (0.41 − 1.45)
Biospecimen_Tissue	0.78 (0.47 − 1.27)
Encounter_ClinicLocation	0.78 (0.47 − 1.27)
Biospecimen_Blood	0.77 (0.46 − 1.25)
FamilyHealthHistory_MentalHealth	0.76 (0.44 − 1.28)
Demographics_Ethnicity	0.76 (0.40 − 1.39)
Demographics_Race	0.73 (0.39 − 1.34)
SexualLife	0.72 (0.47 − 1.08)
BodyMeasurements	0.71 (0.39 − 1.26)
Demographics_Age	0.71 (0.35 − 1.38)
TxProcedure_Other	0.71 (0.42 − 1.16)
SES_Income	0.71 (0.45 − 1.10)
DiseaseCondition_MentalHealth	0.71 (0.41 − 1.18)
Encounter_VisitDate	0.70 (0.42 − 1.15)
Encounter_PhysicianName	0.69 (0.42 − 1.12)
Encounter_MRN	0.69 (0.42 − 1.10)
Demographics_Sex	0.68 (0.34 − 1.32)
TxProcedure_Bariatric	0.68 (0.40 − 1.11)
Demographics_SexualOrientation	0.67 (0.37 − 1.18)
TxProcedure_GenitouriaryReproductive	0.66 (0.39 − 1.09)
TxProcedure_Cosmetic	0.66 (0.39 − 1.09)
ContactInfo_Email	0.63 (0.41 − 0.96)
Medication_OtherMedication	0.62 (0.35 − 1.07)
TxProcedure_MentalHealth	0.61 (0.36 − 1.00)
ContactInfo_Name	0.60 (0.38 − 0.92)
Medication_MentalHealth	0.56 (0.33 − 0.94)
ContactInfo_HomeAddress	0.56 (0.36 − 0.85)
ContactInfo_Phone	0.54 (0.35 − 0.82)

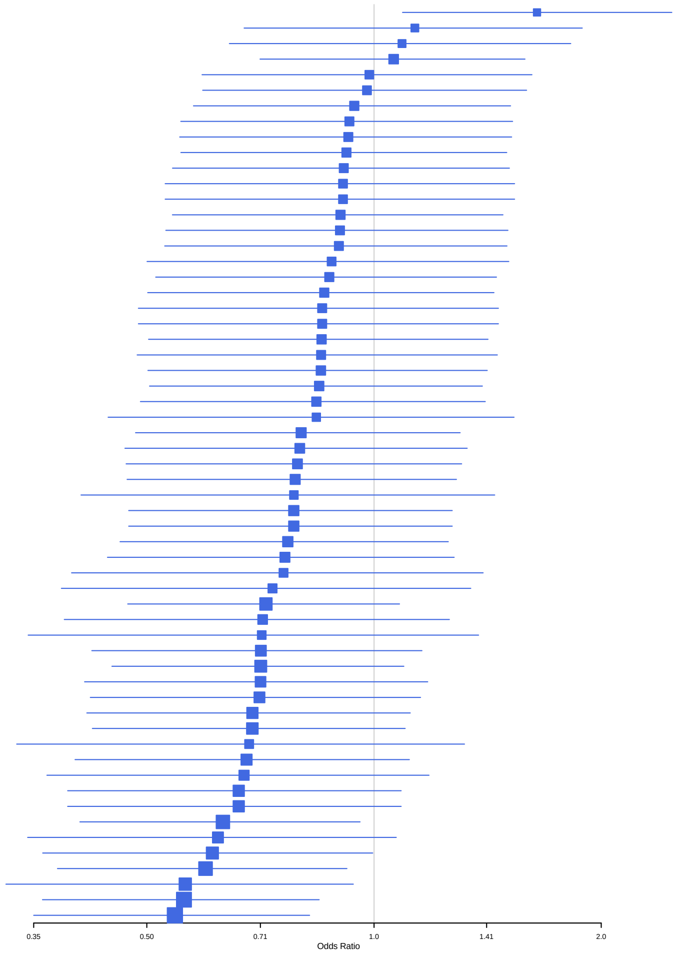

The 59 sharable items were sorted by unadjusted odds ratios and shown with their 95% confidence intervals. For each sharable variable, a 2-by-2 table was constructed using a binary outcome (shared vs. not shared) and a binary exposure variable, gender (female vs. male). Then the odds ratio and its 95% confidence interval were calculated. Row labels are expressed in the format of “Category_Item”. A category with no item was expressed simply as “Category.” Abbreviations: CT, Computerized Tomography; DNA, DeoxyriboNucleic Acid; MRI, Magnetic Resonance Imaging; MRN, Medical Record Number; SES, Social Economic Status; STD, Sexually Transmitted Disease; TxProcedure, Treatment Procedure.

© 2019 Kim J et al. *JAMA Network Open.*

eFigure 11. Forest plot of unadjusted odds ratio for self-reported health (>= very good vs. <very good).

Self−reported health (>= very good vs. < very good)

Variable	Odds Ratio (95% CI)
ContactInfo_Phone	1.44 (1.06 − 1.96)
FamilyHealthHistory_Other	1.42 (0.98 − 2.06)
Biospecimen_Tissue	1.38 (0.95 − 2.00)
ImagingTest_OtherImagingTest	1.36 (0.92 − 2.01)
LabTestResults_GeneticTest	1.36 (0.92 − 2.01)
LabTestResults_Other	1.33 (0.91 − 1.95)
ContactInfo_Email	1.32 (0.96 − 1.81)
FamilyHealthHistory_MentalHealth	1.31 (0.89 − 1.91)
ContactInfo_HomeAddress	1.30 (0.95 − 1.78)
PregnancyHistory	1.29 (0.94 − 1.76)
Biospecimen_Urine	1.28 (0.88 − 1.87)
FamilyHealthHistory_SubstanceAbuse	1.28 (0.88 − 1.86)
FamilyHealthHistory_SexualReproductive	1.28 (0.88 − 1.86)
Encounter_MRN	1.28 (0.89 − 1.84)
TxProcedure_Other	1.26 (0.88 − 1.82)
ImagingTest_X.ray	1.26 (0.83 − 1.91)
DiseaseCondition_MentalHealth	1.25 (0.86 − 1.83)
Medication_MentalHealth	1.24 (0.85 − 1.80)
Biospecimen_Blood	1.24 (0.84 − 1.81)
LabTestResults_STDtest	1.23 (0.84 − 1.82)
TxProcedure_Bariatric	1.22 (0.84 − 1.76)
Medication_OtherMedication	1.21 (0.82 − 1.79)
Encounter_ClinicSpecialty	1.21 (0.83 − 1.77)
LabTestResults_DrugScreening	1.21 (0.82 − 1.78)
ImagingTest_CTscan	1.21 (0.80 − 1.82)
Encounter_PhysicianName	1.20 (0.83 − 1.73)
TxProcedure_GenitouriaryReproductive	1.19 (0.82 − 1.73)
Encounter_ClinicLocation	1.19 (0.82 − 1.73)
LabTestResults_DNAsequencing	1.19 (0.81 − 1.75)
ContactInfo_Name	1.18 (0.85 − 1.64)
Encounter_VisitDate	1.17 (0.81 − 1.70)
AdoptionHistory	1.17 (0.86 − 1.59)
ImagingTest_MRI	1.16 (0.77 − 1.74)
DiseaseCondition_SubstanceAbuse	1.15 (0.78 − 1.67)
DiseaseCondition_SexualReproductive	1.14 (0.77 − 1.68)
TxProcedure_MentalHealth	1.14 (0.79 − 1.64)
DiseaseCondition_Other	1.13 (0.78 − 1.64)
Demographics_Ethnicity	1.13 (0.74 − 1.72)
SexualLife	1.11 (0.81 − 1.51)
SES_Income	1.10 (0.79 − 1.53)
TxProcedure_Cosmetic	1.10 (0.76 − 1.58)
Lifestyle_SocialIsolation	1.04 (0.71 − 1.53)
Demographics_SexualOrientation	1.01 (0.67 − 1.50)
Lifestyle_Drug	1.00 (0.69 − 1.45)
Demographics_Race	1.00 (0.65 − 1.54)
SES_Education	0.99 (0.67 − 1.45)
Lifestyle_Alcohol	0.97 (0.67 − 1.42)
SES_Occupation	0.97 (0.66 − 1.43)
BodyMeasurements	0.97 (0.65 − 1.44)
Allergies	0.96 (0.62 − 1.48)
Lifestyle_Diet	0.96 (0.64 − 1.42)
Lifestyle_Stress	0.96 (0.64 − 1.42)
Lifestyle_Smoking	0.96 (0.65 − 1.39)
SES_MaritalStatus	0.95 (0.64 − 1.42)
VitalSigns	0.94 (0.60 − 1.45)
SES_InsuranceStatus	0.93 (0.64 − 1.35)
Lifestyle_Exercise	0.92 (0.61 − 1.38)
Demographics_Sex	0.88 (0.55 − 1.38)
Demographics_Age	0.84 (0.52 − 1.34)

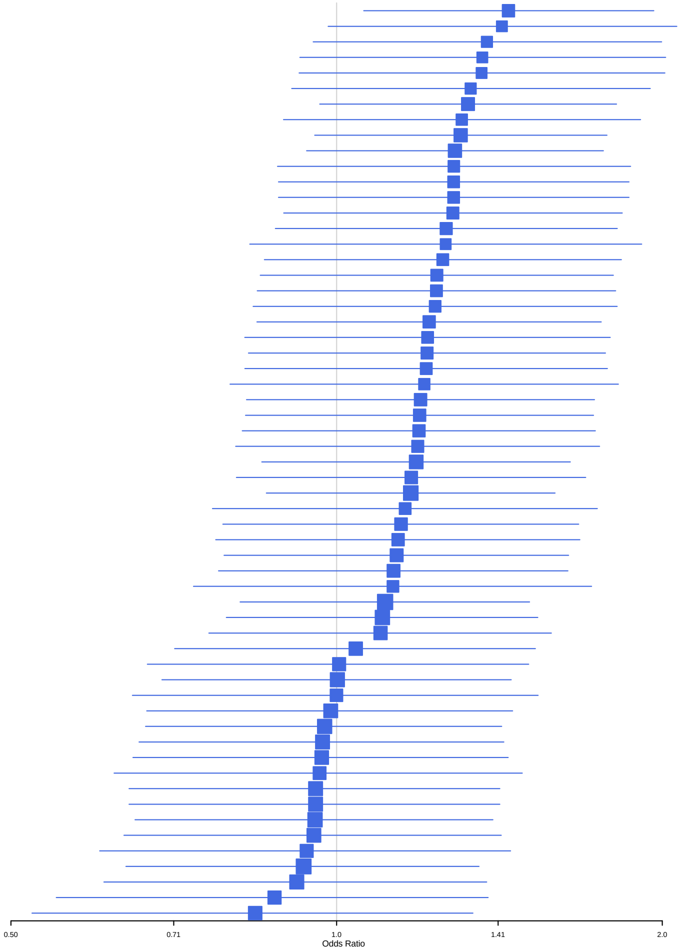

The 59 sharable items were sorted by unadjusted odds ratios and shown with their 95% confidence intervals. For each sharable variable, a 2-by-2 table was constructed using a binary outcome (shared vs. not shared) and a binary exposure variable, self-reported health (>= very good vs. < very good). Then the odds ratio and its 95% confidence interval were calculated. Row labels are expressed in the format of “Category_Item”. A category with no item was expressed simply as “Category.” Abbreviations: CT, Computerized Tomography; DNA, DeoxyriboNucleic Acid; MRI, Magnetic Resonance Imaging; MRN, Medical Record Number; SES, Social Economic Status; STD, Sexually Transmitted Disease; TxProcedure, Treatment Procedure.

© 2019 Kim J et al. *JAMA Network Open.*

eFigure 12. Forest plot of unadjusted odds ratio for race (white vs. non-white).

Race (white vs. nonwhite)

Variable	Odds Ratio (95% CI)
FamilyHealthHistory_SubstanceAbuse	2.35 (1.38 − 3.94)
SES_MaritalStatus	2.29 (1.33 − 3.86)
FamilyHealthHistory_MentalHealth	2.19 (1.26 − 3.71)
SES_Education	2.18 (1.26 − 3.70)
SES_InsuranceStatus	2.15 (1.28 − 3.54)
DiseaseCondition_SubstanceAbuse	2.14 (1.25 − 3.58)
FamilyHealthHistory_Other	2.14 (1.25 − 3.58)
SES_Occupation	2.03 (1.18 − 3.43)
FamilyHealthHistory_SexualReproductive	1.98 (1.14 − 3.37)
TxProcedure_Bariatric	1.96 (1.15 − 3.26)
Biospecimen_Blood	1.96 (1.15 − 3.25)
PregnancyHistory	1.94 (1.23 − 3.03)
TxProcedure_GenitouriaryReproductive	1.92 (1.13 − 3.19)
Biospecimen_Urine	1.92 (1.12 − 3.18)
Biospecimen_Tissue	1.88 (1.10 − 3.11)
LabTestResults_Other	1.87 (1.05 − 3.22)
DiseaseCondition_Other	1.85 (1.07 − 3.13)
LabTestResults_DrugScreening	1.82 (1.02 − 3.14)
BodyMeasurements	1.82 (0.97 − 3.27)
TxProcedure_Other	1.79 (1.05 − 2.99)
TxProcedure_Cosmetic	1.79 (1.04 − 2.98)
TxProcedure_MentalHealth	1.77 (1.05 − 2.93)
Medication_OtherMedication	1.76 (0.98 − 3.07)
SexualLife	1.76 (1.12 − 2.71)
ImagingTest_MRI	1.75 (0.96 − 3.08)
ImagingTest_CTscan	1.75 (0.96 − 3.08)
Encounter_ClinicSpecialty	1.73 (1.00 − 2.91)
Demographics_SexualOrientation	1.72 (0.93 − 3.08)
Demographics_Ethnicity	1.72 (0.87 − 3.24)
LabTestResults_STDtest	1.70 (0.96 − 2.91)
LabTestResults_DNAsequencing	1.70 (0.96 − 2.91)
DiseaseCondition_SexualReproductive	1.68 (0.93 − 2.91)
Demographics_Race	1.67 (0.84 − 3.13)
Encounter_VisitDate	1.66 (0.96 − 2.79)
Encounter_PhysicianName	1.65 (0.97 − 2.74)
ImagingTest_OtherImagingTest	1.65 (0.92 − 2.85)
LabTestResults_GeneticTest	1.64 (0.92 − 2.85)
ImagingTest_X.ray	1.64 (0.89 − 2.92)
Encounter_ClinicLocation	1.63 (0.95 − 2.73)
DiseaseCondition_MentalHealth	1.63 (0.92 − 2.78)
Medication_MentalHealth	1.61 (0.92 − 2.73)
Encounter_MRN	1.58 (0.94 − 2.59)
SES_Income	1.54 (0.94 − 2.47)
Lifestyle_Drug	1.53 (0.87 − 2.59)
Lifestyle_SocialIsolation	1.52 (0.84 − 2.65)
Lifestyle_Smoking	1.51 (0.84 − 2.59)
AdoptionHistory	1.50 (0.94 − 2.34)
Lifestyle_Diet	1.48 (0.80 − 2.62)
Lifestyle_Exercise	1.46 (0.78 − 2.62)
Allergies	1.42 (0.69 − 2.73)
ContactInfo_Email	1.41 (0.88 − 2.22)
Lifestyle_Alcohol	1.41 (0.79 − 2.42)
VitalSigns	1.36 (0.66 − 2.62)
Lifestyle_Stress	1.36 (0.73 − 2.41)
ContactInfo_HomeAddress	1.34 (0.84 − 2.09)
ContactInfo_Phone	1.31 (0.82 − 2.03)
Demographics_Sex	1.28 (0.58 − 2.58)
ContactInfo_Name	1.15 (0.69 − 1.85)
Demographics_Age	1.14 (0.50 − 2.37)

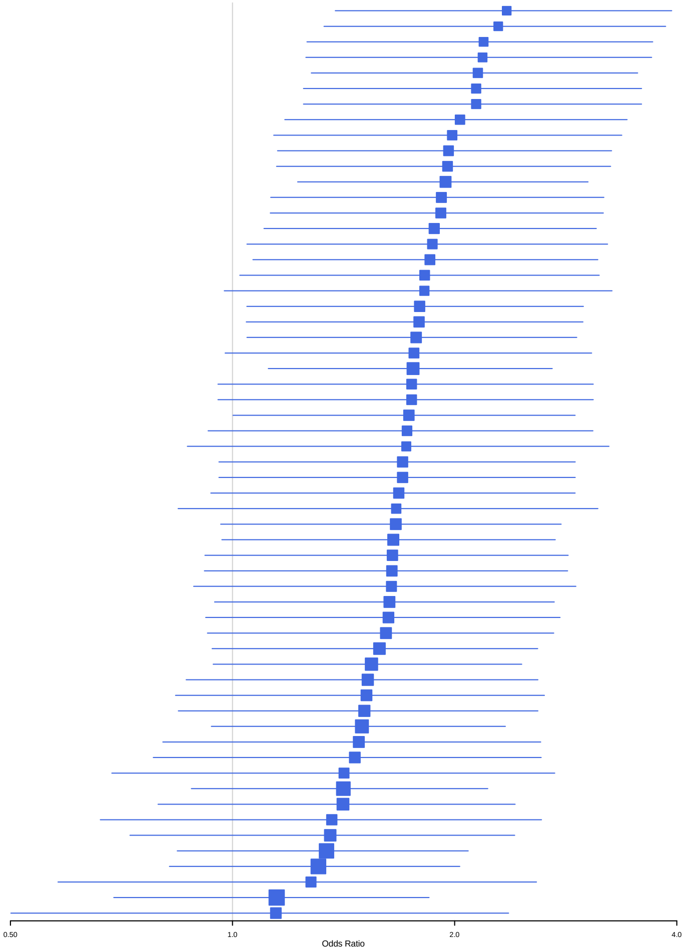

The 59 sharable items were sorted by unadjusted odds ratios and shown with their 95% confidence intervals. For each sharable variable, a 2-by-2 table was constructed using a binary outcome (shared vs. not shared) and a binary exposure variable, race (white vs. nonwhite). Then the odds ratio and its 95% confidence interval were calculated. Row labels are expressed in the format of “Category_Item”. A category with no item was expressed simply as “Category.” Abbreviations: CT, Computerized Tomography; DNA, DeoxyriboNucleic Acid; MRI, Magnetic Resonance Imaging; MRN, Medical Record Number; SES, Social Economic Status; STD, Sexually Transmitted Disease; TxProcedure, Treatment Procedure.

© 2019 Kim J et al. *JAMA Network Open.*

eFigure 13. Forest plot of unadjusted odds ratio for site (#2 vs. #1).

Site (#2 vs. #1)

Variable	Odds Ratio (95% CI)
SexualLife	0.90 (0.65 − 1.25)
SES_MaritalStatus	0.79 (0.51 − 1.21)
Encounter_ClinicSpecialty	0.78 (0.51 − 1.16)
ContactInfo_HomeAddress	0.77 (0.55 − 1.08)
Lifestyle_Smoking	0.77 (0.50 − 1.15)
ContactInfo_Phone	0.75 (0.53 − 1.04)
Encounter_ClinicLocation	0.75 (0.49 − 1.12)
Encounter_PhysicianName	0.74 (0.49 − 1.11)
Encounter_MRN	0.73 (0.49 − 1.08)
ContactInfo_Email	0.73 (0.51 − 1.03)
Lifestyle_Alcohol	0.73 (0.47 − 1.09)
Lifestyle_Exercise	0.72 (0.45 − 1.11)
Demographics_SexualOrientation	0.72 (0.45 − 1.11)
AdoptionHistory	0.72 (0.51 − 1.00)
Lifestyle_Drug	0.71 (0.47 − 1.07)
SES_Income	0.71 (0.49 − 1.02)
Medication_MentalHealth	0.71 (0.46 − 1.07)
ContactInfo_Name	0.70 (0.48 − 1.00)
Lifestyle_SocialIsolation	0.70 (0.45 − 1.06)
SES_InsuranceStatus	0.69 (0.45 − 1.04)
Encounter_VisitDate	0.69 (0.45 − 1.03)
VitalSigns	0.69 (0.42 − 1.10)
Lifestyle_Diet	0.69 (0.44 − 1.05)
Lifestyle_Stress	0.69 (0.44 − 1.05)
Allergies	0.68 (0.41 − 1.08)
DiseaseCondition_MentalHealth	0.68 (0.44 − 1.02)
FamilyHealthHistory_MentalHealth	0.67 (0.43 − 1.02)
TxProcedure_Cosmetic	0.66 (0.44 − 0.99)
Demographics_Race	0.66 (0.40 − 1.06)
Medication_OtherMedication	0.66 (0.42 − 1.01)
FamilyHealthHistory_SexualReproductive	0.66 (0.43 − 0.99)
PregnancyHistory	0.66 (0.46 − 0.93)
LabTestResults_DNAsequencing	0.66 (0.42 − 1.00)
TxProcedure_MentalHealth	0.65 (0.43 − 0.98)
SES_Education	0.65 (0.42 − 1.00)
Biospecimen_Urine	0.65 (0.42 − 0.98)
SES_Occupation	0.64 (0.41 − 0.98)
FamilyHealthHistory_Other	0.64 (0.42 − 0.97)
Demographics_Ethnicity	0.63 (0.38 − 1.01)
FamilyHealthHistory_SubstanceAbuse	0.63 (0.41 − 0.95)
LabTestResults_GeneticTest	0.63 (0.40 − 0.97)
DiseaseCondition_SexualReproductive	0.62 (0.40 − 0.96)
Biospecimen_Blood	0.62 (0.40 − 0.95)
LabTestResults_STDtest	0.62 (0.39 − 0.95)
Biospecimen_Tissue	0.62 (0.40 − 0.93)
BodyMeasurements	0.61 (0.38 − 0.95)
TxProcedure_Other	0.61 (0.40 − 0.92)
ImagingTest_X.ray	0.60 (0.37 − 0.96)
DiseaseCondition_Other	0.60 (0.39 − 0.91)
TxProcedure_Bariatric	0.60 (0.39 − 0.90)
TxProcedure_GenitouriaryReproductive	0.59 (0.38 − 0.89)
ImagingTest_CTscan	0.59 (0.36 − 0.93)
DiseaseCondition_SubstanceAbuse	0.58 (0.37 − 0.89)
ImagingTest_MRI	0.57 (0.35 − 0.90)
Demographics_Age	0.55 (0.31 − 0.93)
ImagingTest_OtherImagingTest	0.54 (0.34 − 0.84)
LabTestResults_Other	0.53 (0.33 − 0.82)
Demographics_Sex	0.53 (0.30 − 0.88)
LabTestResults_DrugScreening	0.52 (0.33 − 0.82)

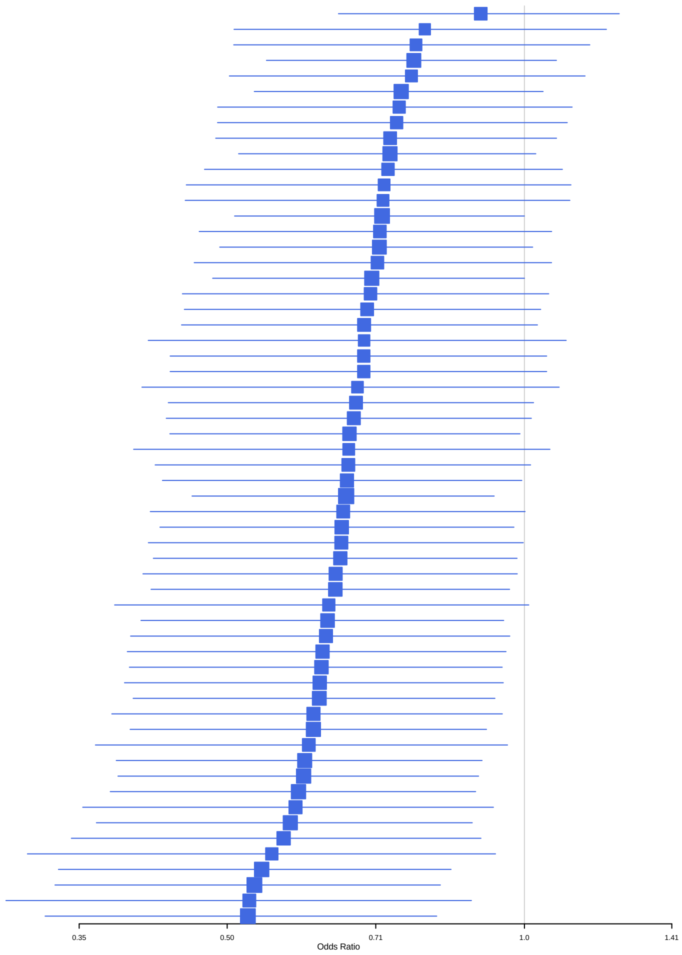

The 59 sharable items were sorted by unadjusted odds ratios and shown with their 95% confidence intervals. For each sharable variable, a 2-by-2 table was constructed using a binary outcome (shared vs. not shared) and a binary exposure variable, site (#2 vs. #1). Then the odds ratio and its 95% confidence interval were calculated. Row labels are expressed in the format of "Category_Item". A category with no item was expressed simply as "Category." Abbreviations: CT, Computerized Tomography; DNA, DeoxyriboNucleic Acid; MRI, Magnetic Resonance Imaging; MRN, Medical Record Number; SES, Social Economic Status; STD, Sexually Transmitted Disease; TxProcedure, Treatment Procedure.

© 2019 Kim J et al. *JAMA Network Open.*
